# Genome-Wide Analyses of Repeat-Induced Point Mutations in the Ascomycota

**DOI:** 10.3389/fmicb.2020.622368

**Published:** 2021-02-01

**Authors:** Stephanie van Wyk, Brenda D. Wingfield, Lieschen De Vos, Nicolaas A. van der Merwe, Emma T. Steenkamp

**Affiliations:** Department of Biochemistry, Genetics and Microbiology, Forestry and Agricultural Biotechnology Institute (FABI), University of Pretoria, Pretoria, South Africa

**Keywords:** repeat-induced point mutation, RIP, Ascomycota, genome evolution, GC content

## Abstract

The Repeat-Induced Point (RIP) mutation pathway is a fungus-specific genome defense mechanism that mitigates the deleterious consequences of repeated genomic regions and transposable elements (TEs). RIP mutates targeted sequences by introducing cytosine to thymine transitions. We investigated the genome-wide occurrence and extent of RIP with a sliding-window approach. Using genome-wide RIP data and two sets of control groups, the association between RIP, TEs, and GC content were contrasted in organisms capable and incapable of RIP. Based on these data, we then set out to determine the extent and occurrence of RIP in 58 representatives of the Ascomycota. The findings were summarized by placing each of the fungi investigated in one of six categories based on the extent of genome-wide RIP. *In silico* RIP analyses, using a sliding-window approach with stringent RIP parameters, implemented simultaneously within the same genetic context, on high quality genome assemblies, yielded superior results in determining the genome-wide RIP among the Ascomycota. Most Ascomycota had RIP and these mutations were particularly widespread among classes of the Pezizomycotina, including the early diverging Orbiliomycetes and the Pezizomycetes. The most extreme cases of RIP were limited to representatives of the Dothideomycetes and Sordariomycetes. By contrast, the genomes of the Taphrinomycotina and Saccharomycotina contained no detectable evidence of RIP. Also, recent losses in RIP combined with controlled TE proliferation in the Pezizomycotina subphyla may promote substantial genome enlargement as well as the formation of sub-genomic compartments. These findings have broadened our understanding of the taxonomic range and extent of RIP in Ascomycota and how this pathway affects the genomes of fungi harboring it.

## Introduction

The Ascomycota represents the largest taxonomic division of the fungal kingdom and includes a vast collection of diverse species (Schoch et al., 2009). These fungi are ubiquitous and can be found in all environmental settings where they are key players in the recycling of organic matter in aquatic and terrestrial systems (Mueller and Schmit, 2007). The group is also notorious for its pathogenic potential and many of them threaten not only human and animal health, but also all forms of plant life (Bowman et al., 1992; Dong et al., 2015). Apart from their medical and economic importance, certain Ascomycota are also exploited for their industrial and commercial properties (de Oliveira and de Graaff, 2011; Grigoriev et al., 2011). Their products are essential in fermentation processes and the production of sought after foods and products, such as meat alternatives (Kumar et al., 2017), biofuels (Grigoriev et al., 2011), organic chemicals (Payne et al., 2015), enzymes, and antibiotics (Martens and Demain, 2017).

The reproductive biology of the Ascomycota is remarkably diverse (Billiard et al., 2012; Wilken et al., 2017). Many reproduce sexually via meiosis to generate progeny in the form of ascospores. In such fungi, maintenance of this ability ensures the generation of genetic diversity and reshuffling of existing variation within populations. Sexual reproduction and meiosis also allow certain members of the phylum use of the Repeat-Induced Point (RIP) mutation pathway for genomic defense against “selfish” or repeated sequence elements (Selker and Garrett, 1988; Selker, 1990). The RIP pathway operates as a type of “quality control” mechanism that ensures integrity of the fungal genome by protecting it from the genetic consequences of these elements, particularly those associated with Transposable Elements (TEs; Cambareri et al., 1991; Selker, 2002). It distinguishes targets based on shared homology between duplicates (Selker and Garrett, 1988), where the targeted regions are permanently mutated by the introduction of cytosine to thymine transition mutations (Cambareri et al., 1991; Watters et al., 1999). Because the essential processes of the pathway are only active after fertilization and immediately before meiosis occurs (Selker and Garrett, 1988; Selker, 1990), RIP is considered to be exclusively available to fungi that are actively recombining.

The RIP pathway evolved early in the evolutionary history of fungi, most likely before the divergence of the subkingdom Dikarya (Horns et al., 2012; Meerupati et al., 2013). RIP has been experimentally and computationally observed only in Ascomycota and certain Basidiomycota (Clutterbuck, 2011; Horns et al., 2012; Hane et al., 2015). The exact taxonomic range of RIP remains ill-defined, but RIP mutation has been recorded in the early diverging lineages of the Pezizomycotina (Meerupati et al., 2013), as well as members of the classes Sordariomycetes and Dothideomycetes (Clutterbuck, 2011).

The study of RIP has allowed important conclusions to be drawn regarding the role of this process in fungal evolution. Apart from maintaining genome integrity, RIP can bring about a range of functionally consequential genetic changes (Rouxel et al., 2011; Meerupati et al., 2013; King et al., 2015; Testa et al., 2016; van Wyk et al., 2019a). This is because RIP activity can lead to the formation of long stretches of AT-rich sequences, causing such genomic regions to be generally gene sparse (Rouxel et al., 2011; van Wyk et al., 2019a). Also, large-scale accumulation of RIP products promotes the loss of sequence similarity between homologous DNA sequences (Ellison et al., 2011; van Wyk et al., 2019a), while RIP-associated methylation brings about suppressed recombination (Ellison et al., 2011). The combined effects of these processes contribute to lineage divergence and lineage-specific evolution, as have been observed in fungi such as *Neurospora tetrasperma* (Ellison et al., 2011; Sun et al., 2017) and species in the *Fusarium fujikuroi* complex (van Wyk et al., 2019a).

Since the discovery of RIP, a range of *in silico* methods have been used to study RIP (Hane and Oliver, 2008, 2010; Horns et al., 2012; Testa et al., 2016; van Wyk et al., 2019b). In these methods, RIP is identified based on the occurrence of extensive directional mutations (i.e., cytosine to thymine transition mutations; Clutterbuck, 2011). Genomic regions affected by RIP display increased frequencies of transition to transversion ratios, and further exhibit reduced GC content (Hane and Oliver, 2008; Clutterbuck, 2011). *In silico* RIP analyses also account for the genetic context of RIP transitions, where RIP detection is dependent on the nucleotide sequences adjacent to those recognized as appropriate RIP targets (Selker et al., 2003). Therefore, the occurrence of cytosine to thymine biased transitions, together with the genetic context of these mutations, provided the theoretical basis for the commonly used RIP indices (Margolin et al., 1998; Selker et al., 2003; Lewis et al., 2009; van Wyk et al., 2019b).

Despite the availability of different *in silico* techniques for studying RIP, little is known about the occurrence and extent of RIP on a genome-wide level for the Ascomycota. This is mainly because of the technical complexities associated with the detection of RIP, which often relies on appropriate alignment of homologous genomic segments, especially those containing TEs and repeated regions (Hane and Oliver, 2008). However, accurate identification of these elements is dependent on the comprehensiveness of existing TE databases, while the elements in question could have undergone extensive degradation since their initial emergence (Hane et al., 2015). Also, similar types of repeats and TEs may not be present in all the taxa investigated, which further hinders the analysis of RIP across a broad taxonomic range. Although sliding window-based approaches have been previously established, these have not been adopted widely due to the extensive occurrence of false positive results associated with the use of overly lenient parameters (Hane and Oliver, 2008; van Wyk et al., 2019b). To exacerbate the matter further, most analyses of RIP are performed within the bounds of gene-level duplications or TE repeats (Hane and Oliver, 2008) and do not take into account the fact that RIP may “leak” beyond the bounds of genetic targets, thereby affecting adjacent genomic regions (Irelan et al., 1994; Rouxel et al., 2011; Van de Wouw et al., 2018; van Wyk et al., 2019a). The consequence of all these complexities and limitations is that our understanding of the extent of RIP on a genome-wide scale remains unclear, because such information cannot be readily extrapolated from TE and gene-level RIP analyses.

This study considered the genome-wide effects of the RIP pathway in the Ascomycota using whole genome sequences available in the public domain. To overcome the typical challenges associated with RIP analyses, we used an alignment-free method, based on a sliding-window approach with optimized RIP index parameters (van Wyk et al., 2019b) rather than single-type TE/repeat sequence matrices (Hane and Oliver, 2008). Our three specific aims were to (i) evaluate the extent to which genome quality might influence the detection of RIP; (ii) establish baseline criteria for recognizing RIP capability in fungal genomes using *in silico* analysis; and (iii) compare the distribution and extent of RIP across the genomes of Ascomycota. The results of this study will provide valuable insights regarding the taxonomic range of RIP and how it affects the genomes of the fungi harboring it.

## Materials and Methods

### Genome Sequences

Publicly available whole-genome sequences were obtained from the database of the National Centre for Biotechnology Information (NCBI). These included sequenced representatives of all subphyla of the Ascomycota, i.e., Pezizomycotina (46 genomes), Saccharomycotina (nine genomes), and Taphrinomycotina (three genomes). The repeat content for each genome was estimated using the REPET pipeline (Quesneville et al., 2005; Flutre et al., 2011). Based on reports from published literature, the fungi in our data set were also assigned as “sexual,” “asexual,” and “unknown” (Supplementary Table S1, and associated references), because RIP is considered to be associated with premeiotic processes (Selker and Garrett, 1988; Selker, 1990). Where relevant, RIP-positive and RIP-negative control genome sequences were included (Supplementary Table S1, as described previously; van Wyk et al., 2019b). These controls included six genomes of organisms that are, respectively, RIP-capable (i.e., *Neurospora crassa*, *Trichoderma reesei*, and *Leptosphaeria maculans*; Selker et al., 2003; Fudal et al., 2009; Li et al., 2017) and not RIP-capable (i.e., *Escherichia coli*, *Encephalitozoon cuniculi*, and *Candida albicans*; Supplementary Table S1).

Representatives for each taxonomic group were chosen based on the quality and completeness of their genome assemblies. Where possible, representatives classified as “complete” or “chromosome” assembly level, according to the NCBI database, with high N50 values were also selected for subsequent analyses (Supplementary Table S1). All taxa investigated were evaluated with the Benchmarking Universal Single-Copy Orthologs (BUSCO) v. 3.0.2 (Simão et al., 2015; Waterhouse et al., 2017) as a further indication of genome completeness using the following data sets: Saccharomyceta, Pezizomycotina, Ascomycota, and Microsporidia.

To investigate how the quality and completeness of a genome assembly influences genome-wide RIP statistics, three versions of the *Fusarium circinatum* genome (strain FSP34) were subjected to RIP analysis (Wingfield et al., 2012, 2018; van Wyk et al., 2019a) and the identification of Large RIP-Affected Regions [LRARs; i.e., RIP-affected regions spanning at least 4,000 base pairs (bp); van Wyk et al., 2019b]. This fungus was originally isolated from pitch canker affected *Pinus radiata* tissue in California (Wingfield et al., 2012) and is maintained at −80°C in the culture collection of the Forestry and Agricultural Biotechnology Institute (FABI; University of Pretoria, Pretoria, South Africa). These three assembly versions represent improvements over time as new technologies and more data were used for determining the genome sequence of this fungus. They are referred to as the 2012, 2018, and 2020 versions of the *F. circinatum* (FSP34) genome assembly (Supplementary Table S1). Additionally, the level of genome completeness of the three *F. circinatum* genome assemblies was verified using the BUSCO tool with the Sordariomyceta data set (Simão et al., 2015; Waterhouse et al., 2017).

### Establishing Baseline Criteria for Recognizing RIP Capability Using *in silico* Analysis

Because no previous studies have set out to quantitatively determine the genome-wide occurrence of RIP among members of the Ascomycota, we first needed to determine a baseline to contrast and distinguish fungi that potentially have RIP and those that do not. To this effect, we considered two approaches. The first was to determine whether a particular fungal genome encoded all of the expected genes associated with RIP-capability, DNA methylation, and RIP directed heterochromatic silencing, and the second was whether it contained RIP-like transition mutations. For this purpose, we used a set of positive and negative control genomes.

To study the presence and distribution of genes associated with the functioning of RIP (based on reports from *N. crassa*; Lewis et al., 2010; Gessaman and Selker, 2017; Gladyshev, 2017; Li et al., 2017; Bewick et al., 2019; van Wyk et al., 2019a; He et al., 2020), BLASTp and tBLASTn searches were performed on the fungi investigated in this study. DNA and protein sequences with sufficient similarity (Expect-value < 1 × 10^−5^) to those described in *N. crassa* were identified using the NCBI non-redundant BLAST database. Although the exact genomic mechanism underlying RIP is still under investigation, the query sequences used in these analyses were the two 5-cytosine methyltransferases RID (RIP deficient) and defective in methylation (DIM-2). In these analyses, we also included six other genes whose products were previously suggested to play a role in processes related to DIM-2-mediated DNA methylation and RIP directed heterochromatic silencing in the Ascomycota. These were the cofactors heterochromatic protein 1 (HP-1), DIM-5, DIM-7, DNA damage binding protein 1 (DDB-1), and Cullin-4 (Cul4; Lewis et al., 2010; Gessaman and Selker, 2017; He et al., 2020).

To study the distribution of RIP, and to evaluate the most informative and suitable method for identifying RIP within fungal genomes, the six control organisms used in this study were subjected to sliding window-based RIP analyses using two tools: RIPCAL (v1.0.4) and The RIPper (Hane and Oliver, 2008; van Wyk et al., 2019b). In addition to full RIP analyses as described previously (van Wyk et al., 2019b), we also used The RIPper to investigate the occurrence of RIP mutations relative to changes in GC content for individual windows of each of the control genome sequences. For this, RIP composite indices and GC content were calculated. In all cases, we used 1,000 bp sliding windows and 500 bp step sizes (van Wyk et al., 2019b).

We also complemented the RIP data generated for the control organisms with information on the distribution of TEs obtained from REPET. For *Le. maculans*, chromosome-sized scaffolds were not available, and the 10 largest scaffolds were investigated. Because *N. crassa* is the model organism for RIP (Gladyshev, 2017), detailed genome-wide RIP analyses were performed on each of its chromosomes (linkage groups) to evaluate the occurrence of RIP in telomeric and centromeric regions. To this end, the frequency of telomeric repeat sequences were determined (Lewis et al., 2009; Wu et al., 2009; van Wyk et al., 2018) using a 100% similarity cut-off value for the “TTAGGG” telomeric repeat sequence. Putative genomic regions corresponding to centromeres were identified based on the approach reported previously (Li et al., 2017; i.e., identification of the longest AT-rich blocks in each *N. crassa* chromosome). To determine the proportion of TEs distributed within *N. crassa* LRARs, REPET annotations, and CLC Genomics Workbench v. 8.0 (CLCbio, Aarhus, Denmark) were used.

For comparative purposes, the information generated using the REPET pipeline for the control organisms were used for alignment-based RIP analyses (Hane and Oliver, 2008). The TE data sets were constructed by aligning TE repeat sequences sharing a high degree of nucleotide similarity to Large Retro-transposon derivative (LARD) TE sequences, which were identified using BLASTn searches in CLC Genomics Workbench (Expect-value < 1 × 10^−5^). The resulting data sets were aligned using the multiple sequence alignment online tool MAFFT (v7.471; Katoh et al., 2019), after which each data set was subjected to alignment-based RIP analyses and dinucleotide frequency analyses using highest GC content consensus implemented in RIPCAL (Hane and Oliver, 2008).

By making use of the control genomes, we also investigated the possibility of erroneous identification of RIP using 100 simulated sequences of 100,000 bp in length. These were generated with the Random DNA sequence tool (Stothard, 2000) and subjected to full RIP analyses as before. A chi-square test was used to evaluate whether the frequency of RIP-positive windows in the simulated data and that of the genome sequences investigated differed significantly (*p* < 0.01). For these analyses, the null expectation was that the total number of RIP-positive windows in a particular genome assembly is the same as in the simulated data set.

### Genome-Wide *in silico* RIP Analysis of the Ascomycota

Whole-genome sequence information of species included in this study (Supplementary Table S1) was subjected to RIP analyses using the RIPper (van Wyk et al., 2019b). These analyses employed the stringent RIP parameters (i.e., RIP product > 1.15, RIP substrate ≤ 0.75, and a RIP composite index > 0) suggested previously (van Wyk et al., 2019b). Possible correlations in our datasets (e.g., genomic proportion containing repetitive sequences vs. those containing RIP mutations) were tested using the Free Statistics and Forecasting Software v1.2.1 (Wessa, 2020).

The results of the occurrence and extent of RIP were summarized, and these data were used to categorize individual genomes in different RIP classes. Class 1 referred to genomes containing no RIP (0.0 to < 0.2%), Class 2 for genomes containing trace levels of RIP (total genomic proportion between 0.2 and <1.0%), Class 3 moderately low RIP (total genomic proportion 1.0 to <5.0%), Class 4 moderate RIP (total genomic proportion 5.0 to <10.0%), Class 5 moderately high RIP (total genomic proportion 10.0 to <20.0%), and Class 6 high RIP (total genomic proportion ≥20.0%). Additionally, we determined the presence and distribution of genes encoding DIM-2 and RID together with the five cofactors associated with DIM-2-mediated DNA methylation across the selected Ascomycota. This was done as described above for the control genomes. Finally, we also evaluated the possibility of erroneous identification of RIP in these fungal genomes using simulated data and chi-squared tests as described above. The results obtained for all these analyses were illustrated with a cladogram reflecting the phylogenetic relationships among the taxa included using their taxon classification. The cladogram was constructed in phyloT using NCBI taxonomic identification numbers and visualized using iTOL v. 3 software (Letunic and Bork, 2016).

## Results

### RIP Analyses, Genome Completeness, and Quality of the Genome Assemblies

To evaluate the influence of assembly quality and genome completeness on the genome-wide RIP statistics obtained for a particular organism, three versions (2012, 2018, and 2020) of the genome assembly for *F. circinatum* isolate FSP34 were used (Table 1). These assemblies differed considerably in size, number of contigs/scaffolds, and BUSCO completeness level. The RIPper-based analyses of these assemblies further showed that the total proportion of each assembly that constituted RIP mutations were 4.0, 4.1, and 6.3%, respectively. In other words, with an improvement in genome quality, more RIP mutations could be detected. The same was also true for the total proportion predicted repeat content (4.6 vs. 8.0% for the 2012 and 2020 assembly versions) and number of LRARs detected (45 vs. 168 for the 2012 and 2020 assembly versions). These data thus show that the effects of RIP in a particular fungal genome would be underestimated if low-quality genome sequences are used in the analysis.

**Table 1:** Genome-wide Repeat-Induced Point (RIP) mutation statistics for different versions of the *Fusarium circinatum* (FSP34) genome assembly.

Genome and RIP statistics	Version of the genome assembly		
	2012	2018	2020
Genome size (bp)	42,457,838	43,949,211	45,100,144
Number of scaffolds/contigs in the genome assembly	4,509	420	28
BUSCO genome completeness (%)^1^	91.1	92.3	98.1
Number of windows investigated	88,515	87,898	90,200
GC content (%) for the assembly	47.3	47.4	46.9
Number of RIP affected windows^2^	3,582	3,628	5,685
RIP-affected genomic proportion (%)^3^	4.0	4.1	6.3
Predicted repeat content (%)^4^	4.6	4.3	8.0
Number of LRARs^5^	45	106	168
Average size (bp) of LRARs	7085.4	9547.2	14975.5
Average GC content (%) of LRARs	22.8	22.2	20.8
Genomic proportion (bp) of LRARs	318,842	1,012,006	2,515,882
Product index value for LRARs^6^	1.6	1.6	1.6
Substrate index value for LRARs^7^	0.3	0.3	0.2
Composite index value for LRARs^8^	1.3	1.3	1.3

1 Benchmarking universal single-copy orthologs (Simão et al., 2015; Waterhouse et al., 2017).

2 Based on RIP index values.

3 Proportion of the genome affected by RIP. Calculated using the total number of windows indicating RIP-positive index values against the total number of windows investigated for the entire genome sequence.

4 Estimated using REPET (Quesneville et al., 2005; Flutre et al., 2011).

5 LRAR, large RIP affected genomic regions. More than 4,000 bp that are consecutively RIP affected.

6 Product index value (TpA/ApT): x > 1.15.

7 Substrate index value (CpA + TpG/ApC + GpT): 0.75 ≥ x.

8 Composite index value [(TpA/ApT) − (CpA + TpG/ApC + GpT)]: x > 0.

Due to the substantial impact of genome quality on detection of the substrates and products of RIP, we attempted to exclude genomes of low-quality (as reflected by high numbers of scaffolds) or low completeness (as indicated by a BUSCO completeness) in our subsequent analyses. Accordingly, we included 59 fungal genomes for the study (Supplementary Table S1). Many of the genomes with high BUSCO completeness values were generally also assembled to “chromosome-level” (according to the NCBI database), or when these were not available, to “scaffold-level” (Supplementary Tables S1 and S2). For example, among the 59 fungal genomes, 46 contained 96–99.7% complete BUSCOs and only 11 contained 91–95% complete BUSCOs. Despite their lower completeness, the latter genomes were included in the analysis because they were classified as complete by NCBI and contained a small number of scaffolds (Supplementary Table S1), suggesting that their reduced BUSCO values might be a consequence of their unique evolutionary histories (Simão et al., 2015).

The remaining three genomes selected had exceedingly low BUSCO completeness scores (Supplementary Tables S1 and S2) and were for *Pichia kudriavzevii* (BUSCO: 80.0%), *Duddingtonia flagrans* (BUSCO: 89.1%), and *Symbiotaphrina buchneri* (BUSCO: 75.6%). They were retained for the current study because the published assembly for *Pi. kudriavzevii* represents the complete genome, sequenced from chromosome telomere to telomere (Douglass et al., 2018). The other two apparently incomplete genomes were included to ensure taxonomic breadth (i.e., representatives from the Orbiliomycetes and Xylonomycetes, respectively).

### Establishing Baseline Criteria for Recognizing RIP Capability Using *in silico* Analysis

**Figure 1:**
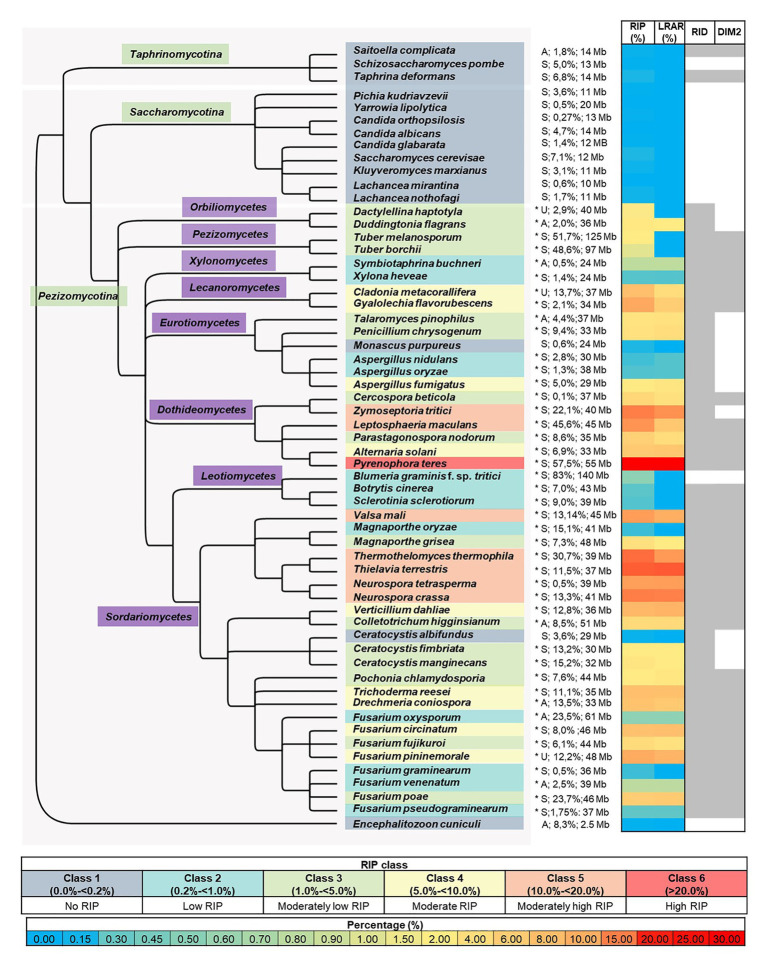
Summary of genome-wide RIP analyses of Ascomycota investigated in this study. From left to right, the first panel represents a cladogram that summarizes the phylogenetic relationships among the fungi investigated, with the microsporidian fungus *En. cuniculi* serving as the root. An asterisk following species names denotes species where a chi-square test indicated that the frequency of RIP-positive windows in the simulated data and that of the actual genome studied differed significantly (*p* < 0.01 confidence level). The panel following the asterisk shows reproductive strategy (“A,” “S,” and “U,” indicating asexual, sexual, and unknown), the predicted proportion (%) of the genome that is repetitive, and genome size [Megabases (Mb)]. The next panel represents a heatmap that illustrates the total proportion (%) of each genome assembly that constitutes RIP mutations and large RIP affected regions (LRARs). The last panel (right) indicates the presence/absence of the RIP-associated genes RID and DIM-2 in each genome (for the latter, see Supplementary Table S3 and Supplementary Figure S1 for detail about these two genes, as well as those for other RIP-associated genes), where shaded columns indicate that a putative homologous sequence identified. The color coding used in the different panels is explained in the keys provided.

For establishing criteria with which to infer whether or not a particular fungus is potentially RIP-capable, we utilized the set of positive and negative control genomes. BLAST analyses against these genomes revealed that the positive controls encoded the two methyltransferases (DIM-2 and RID) demonstrated to mediate the RIP pathway in *N. crassa* (Supplementary Table S3; Figure 1). They also encoded the cofactors associated with DIM-2-mediated DNA methylation (Lewis et al., 2010; Gessaman and Selker, 2017; He et al., 2020), including DIM-5, DIM-7, Cul4, DDB-1, and HP-1 (Supplementary Table S3). None of the negative control fungal genomes encoded a full set of these seven genes.

The RIP statistics generated with The RIPper were consistent with previous work (van Wyk et al., 2019b), where considerable proportions of the positive control genomes had RIP mutations. Although analyses with RIPCAL produced similar results for the RIP-capable fungi, RIPCAL produced spurious results for the negative control genomes. While the statistics generated with The RIPper showed that all three of the negative control genomes were essentially devoid of RIP-like mutations (see below), the RIPCAL data suggested that considerable proportions of these genomes constituted RIP mutations (Supplementary Table S4). As pointed out before (Hane and Oliver, 2008), this discrepancy was likely due to lack of stringency in the parameters used for the sliding window-based RIP analyses tool implemented in RIPCAL.

Overall, the genome-wide RIP statistics generated with The RIPper showed that considerable proportions of the positive control genomes constituted RIP mutations, which was reflected in the number of RIP-affected windows showing high RIP composite index values and reduced GC content (Supplementary Table S4). The negative control organisms remained unchanged by RIP (van Wyk et al., 2019b), with the only exception being the microsporidian genome in which we detected a single RIP-affected window. Another striking difference between these groups was the complete lack of LRARs in any of the negative control organisms as was also observed previously (van Wyk et al., 2019b). Also, when the values for the composite RIP index were considered separate from other RIP index parameters, these values were generally very low and correlated with high GC content (Supplementary Table S4), confirming that they are unlikely to be due to RIP.

**Figure 2:**
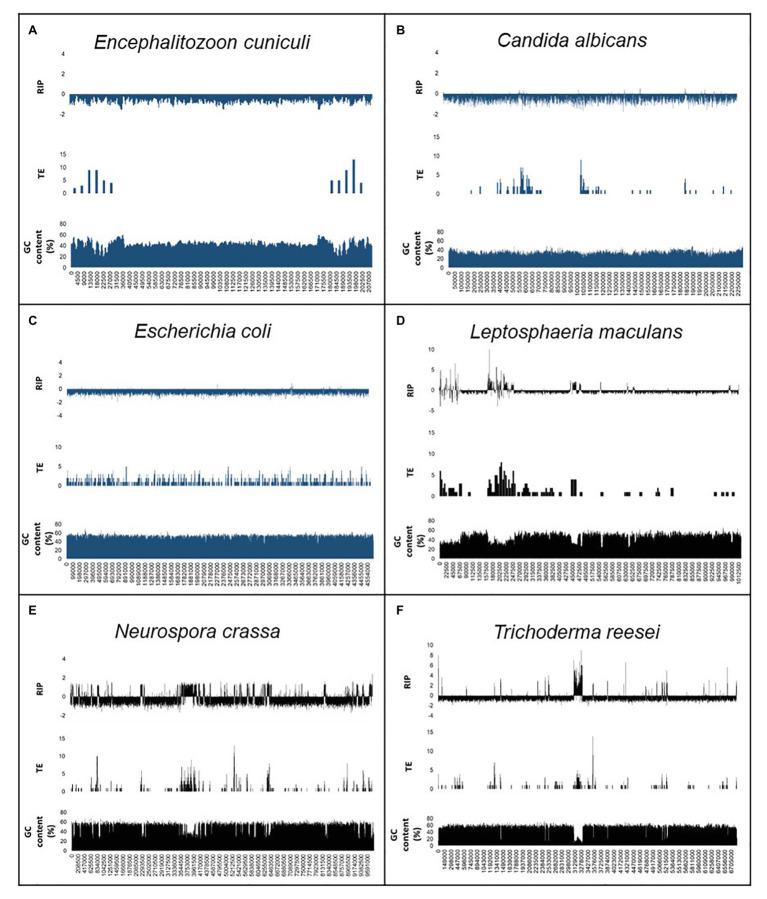
Summary of the genomic features for the RIP-negative **(A–C)** and RIP-positive **(D–F)** control organisms used in this study. The first scaffold or chromosome is indicated for *En. cuniculi* **(A)**, *Ca. albicans* **(B)**, *Le. maculans* **(D)**, *N. crassa* **(E),** and *Tr. reesei* **(F)**, while the whole chromosome of *E. coli* **(C)** are shown. In each panel, the first row shows changes in RIP composite index values, where values > 0 indicate RIP. The second and third rows show the number of transposable elements [TEs; calculated for every 10 and 5 kilo base pair (Kbp) increments] and GC content (calculated for every 1,000 and 500 bp increments). A full list of genome-wide RIP statistics, implementing stringent parameters, is available for these organisms in Supplementary Table S5. Results for the negative control organisms are indicated in blue, and positive control fungi are indicated in black.

We also compared the relationship among TEs and repeated sequences, GC content, and RIP (Figure 2; Supplementary Figure S1). For the positive control fungi, changes in RIP composite index-values corresponded with drastically reduced GC content. This trend was also observed in genomic regions that displayed an increased frequency of TEs and repeats across individual chromosomes or scaffolds of the positive control genomes. By contrast, the distribution of TEs and repeats did not show the same interdependence with RIP composite index values and GC content in the negative control organisms. Notably, the changes in GC content of the negative control organisms remained relatively unchanged across the length of a chromosome or scaffold and did not have regions of extensive GC depletion.

Similar trends were observed when we performed alignment-based and dinucleotide frequency RIP analyses for each of the LARD retrotransposon data sets constructed for the control genome assemblies (Supplementary Figure S1). For the RIP-capable fungi, RIP product, substrate, and composite index values indicated strong RIP responses. This was contrasted to the results obtained for the negative control organisms where changes in RIP index values did not indicate RIP in the TE data sets. Additionally, alignment-based RIP analyses showed that the genomes of RIP-capable fungi were characterized by high frequencies of cytosine to thymine transition mutations, where the dominant RIP mutation forms were CpT to TpT and ApG to ApA RIP transitions. Taken together, RIP was prominent in TE and repeat sequences of fungi known to be RIP-capable and absent in the TEs and repeats of organism not able to undergo RIP. These analyses were thus in agreement with those generated using a sliding-window based approach implemented with The RIPper software. In other words, data generated using the RIPper allow for similar conclusions to be drawn regarding the presence of RIP in a representative assembly, but the RIPper additionally also provides quantitative insight on the extent, and the genomic location of RIP.

**Figure 3:**
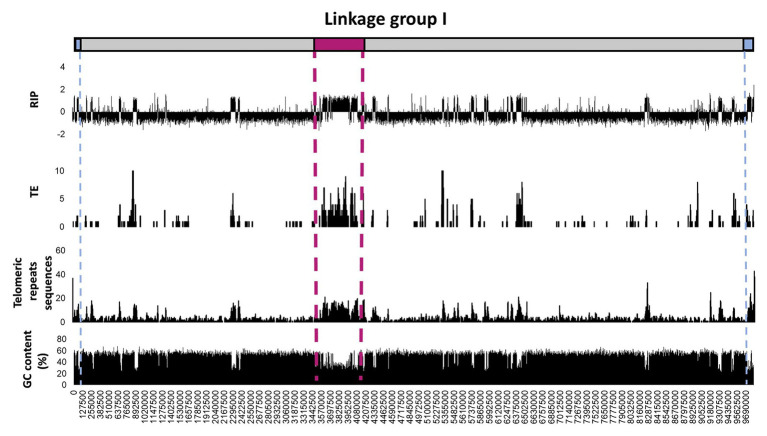
Distribution of RIP mutations, TEs, telomeric repeats, and GC content across the first linkage group/chromosome of *Neurospora crassa*. The putative positions of telomeres and the centromeres are shown in blue and pink, respectively. The RIP graph depicts changes in the RIP composite index across the length of the chromosome, where values above 0 are indicative of RIP. Only the number of TEs or TE-related repeats larger than 100 base pairs (bp) are shown and were calculated using 10 Kbp windows with 5 Kbp increments. The telomeric repeat (TTAGGG/CCCTAA) frequency was calculated for every 10 and 5 Kbp increments. Changes in GC content were determined using a 1,000 bp window and 500 bp increments. See Supplementary Figure S2 for corresponding information for the remainder of the linkage groups/chromosomes of this fungus.

To further investigate regional variation in the occurrence and extent of RIP across the length of a chromosome, detailed RIP analyses were performed for the *N. crassa* chromosome assemblies (Figure 3; Supplementary Figure S2). The most extensively RIP-affected regions of the *N. crassa* chromosomes corresponded to that of the putative centromeric regions (Li et al., 2017). In addition, RIP- and TE-associated repeats were particularly prominent toward the ends of the chromosome, which generally corresponded to an overall increase in the density of telomeric repeat sequences. Therefore, regional variation in RIP across the length of the *N. crassa* chromosomes was common in telomeric and centromeric regions of chromosomes, as well in regions with TEs and repeated sequences. Additionally, a substantial proportion (69.9%) of the predicted TEs and TE repeated sequences were located within the LRARs.

Finally, we evaluated the possibility of erroneously identifying RIP in a particular fungal genome by contrasting the genome-wide RIP statistics obtained with those for simulated data. In the simulated data sets, the frequency of windows with RIP was 0.095 and the frequency of windows without RIP was 99.905. However, for the positive control genomes, the frequencies of windows with RIP were 6.5, 11.8, and 14.5, while those without RIP were 93.5, 88.2, and 85.5 for *Tr. reesei*, *N. crassa*, and *Le. maculans*, respectively. Based on chi-square tests of independence, we thus rejected the null expectation that the frequencies for windows with and without RIP is the same as for the pattern seen with the simulated data sets, at a 99.99% confidence level (*p* < 0.01) for each of the positive control genomes (Supplementary Table S5). However, the null expectation could not be rejected for the negative control *En. cuniculi* (Figure 1; Supplementary Table S5) suggesting that the one window containing RIP occurred by chance and was not a consequence of the RIP activity.

Based on the data generated using the set of control genomes, we considered a RIP-capable fungus as one in which RIP-like mutations constitute >0.2% of its genome and the majority of RIP-positive windows have composite RIP index values higher than 1. The genomes of RIP-capable fungi are expected to also contain LRARs. Significant features of a genome containing RIP has correspondence between areas containing RIP mutations and reduced GC content compared to that of the remainder of the genome. For RIP-capable fungi, we further expect RIP to be a prominent feature of genomic regions rich in TE and other repeated sequences. Also, the genomes of such fungi would likely also maintain the RIP-associated genes, especially those encoding either or both of the 5-cytosine methyltransferases, RID, and DIM-2 (Gladyshev, 2017).

### Genome-Wide *in silico* RIP Analysis of the Ascomycota

BLAST analyses with the sequences of the two methyltransferases known to mediate the RIP pathway, revealed that most of the genome assemblies for Pezizomycotina contained sequences with an average of 44 and 58% similarity to, respectively, DIM-2 and RID from *N. crassa* (Supplementary Table S3; Figure 1). The only exceptions were *Blumeria graminis* f. sp. *tritici* lacking a copy of both genes, and representatives of the Orbiliomycetes, Ceratocystidaceae, and *Zymoseptoria tritici* lacking sequences with similarity to DIM-2. Two species from the Taphrinomycotina (i.e., *Saitoella complicata* and *Taphrina deformans*) also contained both genes. Genes encoding DIM-2 and RID were not detected in any of the other genomes, including all of the Saccharomycotina and *En. cuniculi*.

Regarding the cofactors associated with DIM-2-mediated DNA methylation (Lewis et al., 2010; Gessaman and Selker, 2017; Bewick et al., 2019; He et al., 2020), most of the genomes contained two or more of the five genes examined (Supplementary Table S3). All had sequences similar to those for DIM-5 and Cul4. However, all representatives of the Saccharomycotina lacked sequences for DDB-1 and HP-1, while *Bl.* f. sp. *tritici*, was notable as the only representative of the Pezizomycotina lacking HP-1. In terms of DIM-7, genomes of *En. cuniculi*, Taphrinomycotina, Saccharomycotina, and 12 species in the Pezizomycotina (i.e., all representatives of Orbiliomycetes, Pezizomycetes, Leotiomycetes, and the Eurotiomycetes excluding *Penicillium chrysogenum*) lacked sequences encoding this cofactor.

To investigate the occurrence and extent of RIP in the genomes of Ascomycota, all of the genomes included in this study were subjected to genome-wide RIP analyses using The RIPper’s sliding-window approach (Table 2; Supplementary Table S5; Figure 1). Plotting the results on a cladogram reflecting known phylogenetic relationships (McLaughlin et al., 2009) revealed that these fungi vary greatly in the total proportion of their genomes affected by RIP. While many harbored no evidence of RIP, others were rich in RIP. Among the Sordariomycetes and Dothideomycetes, some were highly affected by RIP, with the most extreme case being *Pyrenophora teres* where RIP constituted 29.53% of its genome. Genomes affected by RIP were also rich in LRARs (Figure 1). In those that were highly RIP-affected, many LRARs were recorded (e.g., as many as 439 LRARs in *Z. tritici*). In such cases, LRARs also constituted considerable proportions of the genomes examined, e.g., >16 Mbp of the 32.7 Mbp. genome of *Py. teres*. These LRARs further also corresponded to regions with reduced GC content (Table 2).

**Table 2:** A summary of genome-wide RIP statistics of representatives of each RIP class and control genome assemblies investigated in this study.

Species	Number of RIP-positive windows^1^	RIP class	RIP-affected genomic proportion (%)^2^	Large RIP affected regions (LRARs)			
				Number detected^3^	Average size (bp)	Average GC (%)	Combined size (bp)
***Candida albicans***	0	1	0.0	0	–	–	–
***Escherichia coli***	0	1	0.0	0	–	–	–
***Encephalitozoon cuniculi***	1	1	0.0	0	–	–	–
*Ceratocystis albifundus*	28	1	0.1	0	–	–	–
*Fusarium graminearum*	206	2	0.3	2.0	6500.0	23.2	13,000
*Fusarium poae*	4,472	3	4.8	182	10821.4	14.9	1,969,500
***Trichoderma reesei***	4,551	4	6.5	179	10662.0	15.6	1,908,500
*Verticillium dahlia*	52 83	4	7.3	104	23966.4	25.1	2,492,500
***Leptosphaeria maculans***	10,225	5	11.8	275	7490.5	32.0	2,059,893
*Zymoseptoria tritici*	10,942	5	14.5	439	9670.0	15.2	4,245,131
***Neurospora crassa***	11,915	5	14.5	435	12347.1	28.4	5,371,000
*Pyrenophora teres*	32,740	6	29.5	406	38886.4	33.0	15,787,878

Organisms highlighted in bold served as control species. A full list of genome-wide RIP statistics is listed in Supplementary Table S5.

1 Based on RIP index values.

2 Proportion of the genome affected by RIP. Calculated using the total number of windows indicating RIP-positive index values against the total number of windows investigated for the entire genome sequence.

3 LRAR, large RIP affected genomic regions. More than 4,000 bp that are consecutively RIP affected.

Overall, large variations were observed in terms of overall repeat content and genome size relative to RIP mutation content across the taxa examined (Figure 1; Supplementary Table S5). Although genome size was moderately correlated with RIP mutation content (Spearman’s rank correlation coefficient *r_s_* = 0.63, *p* < 0.001), there were a number of instances of large genomes having very little RIP and small genomes rich in RIP mutations. For example, 1.5% of the 29 Megabases (Mb) of the *As. fumigatus* genome is affected by RIP, while 0.55% of the 140 Mb *Bl. graminis* f. sp. *tritici* genome constitute RIP mutations. RIP mutation content was also moderately correlated to repeat content estimated with REPET (*r_s_* = 0.52, *p* < 0.001), but there were also numerous outliers. For example, only 0.65% of the *Fusarium oxysporum* genome with its high (23.5%) repeat content constitutes RIP mutations, while the *N. tetrasperma* genome is characterized by a RIP mutation content of 10.3% and repeat content of only 0.5%.

To simplify comparisons among the fungi examined, we recognized six classes of RIP (Figure 1). Fungi in Class 1 were those containing no evidence of RIP [i.e., no/few RIP-positive windows, where RIP constitute small/negligible proportions of the genome (0 to <0.2%)]. Class 6 fungi were those with genomes that are very highly affected by RIP (i.e., >20% of the genome constituting RIP). The impact of RIP on the genomes of Classes 2–5 fungi were recorded as low (>0.2 to <1% RIP), moderately low (>1 to <5% RIP), moderate (>5 to <10% RIP), and moderately high (>10 to <20% RIP), respectively.

The 15 Class 1 genomes included all the Taphrinomycotina and Saccharomycotina investigated, as well as *En. cuniculi*. Overall, the RIP statistics generated for the Taphrinomycotina and Saccharomycotina were similar to those obtained for the negative control organisms (Supplementary Table S4). Among the Pezizomycotina, this was also observed for *Monascus purpureus* in the Eurotiomycetes and *Ceratocystis albifundus* in the Sordariomycetes. In these cases, no or only a few (1–70) RIP windows were detected, and these constituted very small proportions of the overall genome assemblies (0–0.14%). Compared to the remainder of these genomes, the RIP-positive windows typically had low scoring RIP composite index values, and GC content was generally not reduced (Supplementary Table S5).

Twelve fungi were categorized as Class 2 as their genomes contained some LRARs (6–13) and low numbers of RIP-positive windows (180–798), constituting 0.37–0.65% of the individual genomes. These included members from the Xylonomycetes (*Sy. buchneri* and *Xylona heveae*), Sordariomycetes (*Fusarium graminearum*, *Fusarium pseudograminearum*, *F. oxysporum*, and *Fusarium venenatum*), Leotiomycetes (*Botrytis cinerea*), and Eurotiomycetes (*Magnaporthe oryzae*, *Bl. graminis* f. sp. *tritici*, and *Aspergillus nidulans*).

Of the 27 Classes 1 and 2 fungi, the genomes of six (i.e., *Mo. purpureus*, *Sy. buchneri*, *Pi. kudriavzevii*, *Sa. complicata*, *Schizosaccharomyces pombe*, and *Ta. deformans*) had low quality and were fragmented with lower BUSCO completeness scores. Five of these (i.e., *Sy. buchneri*, *Pi. kudriavzevii*, *Sa. complicata*, *Sc. pombe*, and *Ta. deformans*) were all from the Taphrinomycotina and Saccharomycotina in which RIP is also largely absent. However, in the case of *Mo. purpureus* from the Pezizomycotina, more research is needed to determine whether the low repeat and RIP mutation content estimated were artifacts of the quality of its genome.

The remaining 32 genomes all contained substantial evidence of RIP and belonged to RIP Classes 3–6. Sixteen of these were Class 3 and were recorded among the Orbiliomycetes, Pezizomycetes, Eurotiomycetes, Dothideomycetes, and Sordariomycetes (Figure 1). RIP constituted between 1.03 and 4.81% of the genomes of these fungi and, except for *Tuber borchii*, and *Tuber melanosporum*, all had LRARs (17–183 per genome) with highly reduced GC contents and high scoring RIP composite index values (Supplementary Table S5).

Repeat-Induced Point Class 4 included eight fungi (i.e., *Alternaria solani*, *Drechmeria coniospora*, *F. circinatum*, *Cladonia metacorallifera*, *Tr. reesei*, *Verticillium dahlia*, *Fusarium pininemorale*, and *Gyalolechia flavorubescens*) from four taxonomic classes (Eurotiomycetes, Leotiomycetes, Dothideomycetes and Sordariomycetes) of the Pezizomycotina. These fungi had moderate levels of RIP (5 to 9% RIP), and all of them had LRARs (81–182) that constituted a considerable proportion of the individual genomes (2.1 to 6.9% of the genome).

Lastly, seven genomes were placed in Class 5, while one was placed in Class 6. These genomes were from the Dothideomycetes (*Z. tritici*, and *Le. maculans*) and Sordariomycetes (*N. tetrasperma*, *Valsa mali*, *N. crassa*, *Thermothelomyces thermophila*, and *Th. terrestris*) and had high levels of RIP, which constituted more than 10% of the respective genome assemblies. Also, more than 10% of their genomes were predicted to be repetitive. RIP Class 6 was represented by *Py. teres* as its genome was highly affected by RIP (29.53% RIP). The Classes 5 and 6 fungi all had many LRARs (289–406) that constituted large proportions (9.6–28.5%) of the individual genome assemblies.

The results of chi-square tests of independence using a range of simulated data sets showed that our RIP categories were robust. In the case of the Class 2–6 genomes, these tests confirmed that the RIP distribution patterns obtained were not due to chance (Figure 1; Supplementary Table S5). For all the Class 2–6 genomes, the null expectation that the frequency of windows with RIP in a particular genome is the same as in the simulated data sets was rejected at a 99.99% confidence level (*p* < 0.01). As expected, this was not possible for any of the Class 1 genomes where the frequency of windows with RIP did not differ significantly from those recorded in the simulated data.

None of the Class 1 fungi encoded for a full complement of genes associated with the RIP pathway (Supplementary Table S3). The genomes of all Class 1 fungi, except *Ce. albifundus*, lacked a detectable DIM-7 gene, and most lacked one or both of the methyltransferases, RID and DIM-2. This was also the case for the Class 2 genomes of *Bl. graminis* f. sp. *tritici* (lacking HP-1, RID, DIM-2, and DIM-7) and *Aspergillus oryzae* (lacking DIM-2), as well as *As. nidulans*, *Sy. buchneri*, *Bo. cinerea*, and *Sclerotinia sclerotiorum* (all lacking DIM-7). The remaining Class 2 genomes contained both methyltransferases and the cofactors known to be involved in the DIM-2-mediated DNA methylation (i.e., *F. oxysporum*, *F. graminearum*, *F. pseudograminearum*, *F. venenatum*, *Ma. oryzae*, and *X. heveae*). The Class 3 genomes also contained all five DIM-2-associated cofactors, except for DIM-7 that was absent from Orbiliomycetes, Pezizomycetes, *Cl. metacorallifera*, and *Parastagonospora nodorum*. Further, DIM-2 was absent from *Ta. pinophilus*, *Pe. chrysogenum*, *Ce. manginecans*, and *Ce. fimbriata*. Both Class 3 Orbiliomycetes *Da. haptotyla*, and *Du. flagrans* lacked DIM-2 and DIM-7. The Class 4–6 fungi contained all RIP-associated sequences investigated in this study. The only exception was *As. fumigatus* (Class 4), lacking DIM-2. The Class 5 Dothideomycetes species *Z. tritici* was notable as it lacked a complete copy of DIM-2 despite having a large proportion of its genome that constitutes RIP (Dhillon et al., 2010).

Based on our survey of the literature, the fungi in Classes 4–6 are capable of sexual reproduction (Wickerham et al., 1970; Sutton, 1982; Goto et al., 1987; Briza et al., 1990; Raju and Perkins, 1994; Gordon and Martyn, 1997; Keller et al., 1997; Britz et al., 1998; Harrington and Wingfield, 1998; Davis and Smith, 2001; Galagan et al., 2003; Dean et al., 2005; Rau et al., 2005; Rouxel and Balesdent, 2005; Paoletti et al., 2007; Rossi and Languasco, 2007; Tavanti et al., 2007; TeBeest et al., 2007; Bentley et al., 2008; Alby et al., 2009; Seidl et al., 2009; Mestre et al., 2010; Pereira et al., 2011; Rubini et al., 2011; Kurtzman and Robnett, 2012; Wada et al., 2012; Böhm et al., 2013; Studt et al., 2013; Short et al., 2014; Terhem and van Kan, 2014; Marin-Felix et al., 2015; Meng et al., 2015; Belfiori et al., 2016; Cowger et al., 2016; Doughan and Rollins, 2016; Gazis et al., 2016; Lee et al., 2016; Pelin et al., 2016; Son et al., 2016; Evans and Kirk, 2017; Manan, 2017; Randlane et al., 2017; Vaghefi et al., 2017; Vanheule et al., 2017; Yin et al., 2017; Yu et al., 2017; Carreté et al., 2018; Chi et al., 2019; Suffert et al., 2019; Wang et al., 2019a,b; Wu et al., 2020). Apart from a few instances where sexual stages have not been reported (indicated with “U” in Figure 1), the only exception was the nematophagous fungus *Dr. coniospora* that apparently lack a sexual cycle completely (Zhang et al., 2016). In addition to sexual species, Classes 2 and 3 contained various fungi thought to be reproducing mainly asexually. The apparently asexual Class 3 fungi included the nematode-trapping species *Du. flagrans* (Wang et al., 2019b), the plant pathogen *Colletotrichum higginsianum* (O’Connell et al., 2012), and the mycoparasite *Talaromyces pinophilus* (Yilmaz et al., 2014). Class 2 fungi that are apparently asexual included the insect symbiont *Sy. buchneri* (Martinson, 2020), *F. venenatum* used in the production of mycoprotein (Leslie and Summerell, 2006), and the plant pathogen *F. oxysporum* (Braumann et al., 2008).

## Discussion

Genome-wide RIP data have allowed important insights on the taxonomic distribution of RIP in the Ascomycota. Our results provide evidence of RIP in most of the fungal sequences investigated. Extensive levels of RIP were detected in species such as *Tr. reesei*, *N. crassa*, *Le. maculans*, *Th. terrestris*, *Py. teres*, and *Z. tritici*. Fungi with extensive RIP often also were capable of sexual reproduction, had genomes rich in repetitive elements, and encoded the full complement of RIP-associated genes and genes associated with DNA methylation and RIP directed heterochromatic silencing. These findings were comparable to previous *in silico* studies on RIP (Clutterbuck, 2011; Meerupati et al., 2013; Testa et al., 2016; Li et al., 2017; van Wyk et al., 2019a). However, we present for the first-time evidence of RIP in *F. pininemorale*, *X. haveae*, and in representatives of the Lecanoromyces, *G. flavorubescens* and *Cl. metacorallifera*. Surprisingly, RIP was also recorded in a member of the Pezizomycetes, i.e., *Tu. melanosporum*, for which previous *in silico* studies (Clutterbuck, 2011; Chen et al., 2014; Hane et al., 2015) failed to detect RIP in its TE sequences. This may be because this fungus is no longer RIP-capable, and therefore RIP is absent from newly acquired TEs (Clutterbuck, 2011). The latter would be consistent with ancestral RIP activity in this species (Braumann et al., 2008), or the RIP-like mutations observed could have been the result of other mutational processes such as methylation induced premeiotically (MIP; Clutterbuck, 2011).

The patterns of genome-wide RIP observed for the Ascomycota support previous proposals regarding the evolutionary history of RIP. Initial emergence of RIP is thought to predate divergence of the Dikarya (Horns et al., 2012) between 500 and 650 million years ago (Lucking et al., 2009). However, we observed no evidence of RIP in the genomes of the Saccharomycotina and Taphrinomycotina, which is consistent with the notion that RIP capability was lost (Clutterbuck, 2011; Meerupati et al., 2013; Hane et al., 2015) in the ancestral lineages of these fungi following their divergence from the Pezizomycotina during the Paleozoic era (Prieto and Wedin, 2013). In comparison, RIP was particularly prominent among representatives of the Pezizomycotina, and we detected it in at least one representative of each taxonomic class in this subphylum. Additionally, RIP was recorded in all examined representatives of the two most basal groups of the Pezizomycotina, the Orbiliomycetes, and the Pezizomycetes (Spatafora et al., 2006). These data thus suggest that RIP is an ancient fungal genome defense strategy that has remained conserved for hundreds of millions of years across a broad taxonomic range.

Repeat-Induced Point mutations detected in apparently asexual fungi probably also represents signatures of ancestral RIP activity. Neither *F. venenatum* nor *F. oxysporum* have a known sexual stage (Leslie and Summerell, 2006; Braumann et al., 2008), but 0.86 and 0.65% of their respective genomes constituted RIP. This was also true for *Dr. coniospora*, in which 6.01% of its genome represented RIP, despite apparently lacking a sexual stage (Zhang et al., 2016). These findings thus suggest that RIP may be maintained for prolonged periods of time over numerous asexual generations. Alternatively, these fungi may have reproduced sexually in their evolutionary past or may maintain the ability to reproduce sexually but do so cryptically (Braumann et al., 2008). Similarly, the observed RIP in the *Tu. melanosporum* genome most likely reflects ancestral RIP signatures that was acquired before loss of this process, as RIP is notably absent from newly acquired TEs and the genome also lacks LRARs (Clutterbuck, 2011; Chen et al., 2014; Hane et al., 2015). However, the possibility that RIP might be associated with processes other than sexual reproduction cannot be discounted. For example, recent studies on Dothideomycetes suggested the continued functioning of DIM-2 during mitotic cycles (Möller et al., 2020), but whether the DIM-2 activity is dependent on meiosis-linked signals or processes remains unclear.

Our results suggest that loss of RIP in the Saccharomycotina and Taphrinomycotina was likely associated with the loss of genes underlying the RIP pathway. Neither of these fungal groups encode for the full complement of the genes known to be involved in RIP, particularly the two methyltransferases, RID and DIM-2. Such losses probably also explain the lack of RIP capability in some of the more recently evolved lineages of the Pezizomycotina. For example, *Bl. graminis* f. sp. *hordei* is incapable of employing the RIP pathway as a genome defense mechanism due to the loss of key RIP genes (Spanu et al., 2010; Seidl and Thomma, 2017). This is consistent with the results of the current study, which showed that the genome of *Bl. graminis* f. sp. *tritici*, a taxon related to *Bl. graminis* f. sp. *hordei*, lack RID and DIM-2, and displayed low levels of RIP (0.55% of the genome constituted RIP; Class 2). RIP recorded in the *Bl. graminis* f. sp. *tritici* genome is most likely represented remnants of ancestral RIP (Braumann et al., 2008) that were acquired prior to the loss of RIP-associated genes.

Despite not maintaining all the genes underlying the RIP pathway, the genomes of some Ascomycota are rich in RIP mutations. A notable example is *Z. tritici* with its Class 5 genome characterized by extensive RIP in the absence of DIM-2-mediated DNA methylation (Dhillon et al., 2010). As previously speculated (Lorrain et al., 2020; Möller et al., 2020), such instances may reflect recent losses of DIM-2. However, studies on *Metarhizium* showed that RID and DIM-2 exert an additive effect on DNA methylation (Wang et al., 2017), and the functioning of these two pathways might thus influence RIP in a comparable fashion, where the loss of one of these pathways reduces RIP activity instead of completely abolishing it. Indeed, experimentally induced null mutations of either RID or DIM-2 resulted in reduced RIP activity in *N. crassa* (Freitag et al., 2002). Further, RID or DIM-2 mediated RIP may be individually dispensable (Gladyshev, 2017), allowing for occurrence of RIP in fungi where either one of the pathways are no longer maintained.

The extent of RIP differed extensively among the Ascomycota investigated. This was notable in the density of RIP that differed among closely related lineages, regardless of whether the fungi encoded similar sets of RIP-associated genes. For example, the *Ce. albifundus* genome contained no significant evidence of RIP, despite higher RIP levels being recorded in its close relatives *Ce. manginecans* and *Ce. fimbriata* (Class 3). Another example is *Fusarium* where species differed vastly in the extent of RIP in their genomes, despite all containing a full complement of RIP-associated genes. For these fungi, three classes of RIP were recorded: Class 2 for *F. oxysporum*, *F. pseudograminearum*, *F. graminearum*, and *F. venenatum*, Class 3 for *Fusarium poae* and *F. fujikuroi*, and Class 4 for *F. circinatum* and *F. pininemorale*. The differences in the extent of RIP can be explained by differences in genomic content of TEs and repeats, reproductive strategies employed, complexities that arise during meiosis and mating, and/or variation in expression levels of the RIP genes (Hane et al., 2015). In contrast, all representatives of the order Sordariales (*N. crassa*, *N. tertasperma*, *Thi. terrestris*, and *The. thermophila*) had similar genome-wide RIP statistics, where a high level of RIP activity might represent a conserved and important heritable trait.

Our results suggested that TE integration into the host’s genome and its subsequent response *via* RIP causes localized regions with biased sequence composition. For *N. crassa*, previous genome analyses revealed regional variation in RIP across the length of chromosomes, for which the total proportions of RIP can vary greatly (van Wyk et al., 2019b). Here, we complemented these data by showing that RIP is prominent in TE-rich regions of *N. crassa* and other fungi, as well as that centromeric and telomeric genomic regions are TE-rich with substantial levels of RIP. We also showed that RIP content generally coincided with regions of drastically reduced GC content, coupled with a higher frequency of TEs and repeated sequences. This is because TEs and other repeated sequences induce RIP (Cambareri et al., 1991), and extensive RIP directs localized GC-depletion, which is absent from the genomes of fungi that are not RIP-capable (Testa et al., 2016). The combined effects of TE integration and RIP likely also contribute to the formation of LRARs or long stretches of GC depleted genomic regions that are rich in RIP mutations. In fact, the occurrence of LRARs was recently shown to be a good indicator of extensive RIP (Lorrain et al., 2020). Similarly, extensive LRARs typically reflected high levels of RIP, and high repeat contents in the genomes investigated. Nevertheless, these data suggest that RIP directs biased sequence composition, maintained over large genomic regions, which constitutes considerable proportions of the overall genomes of many Ascomycota.

The combined effects of TE integration and RIP activity may also influence the genomic organization of Ascomycota (Frantzeskakis et al., 2019). One way in which these activities could exert their influence is in genome size. For example, fungi that have lost RIP more recently (e.g., *Tu. melanosporum* and species of *Blumeria*) are characterized by substantially enlarged genomes, which are the likely consequence of high levels of TE integration and uncontrolled transposition activity (Payen et al., 2016; Müller et al., 2019). In comparison, fungi from the Taphrinomycotina and Saccharomycotina that lost the ability to employ RIP much earlier presumably rely on other genome defense mechanisms such as MIP or meiotic silencing of unpaired DNA to maintain their more compact and smaller genomes (Gladyshev, 2017). Another way in which TEs and RIP activity can influence the genomic organization of Ascomycota is by driving or contributing to “genomic compartmentalization.” Notable examples are *Ve. dahlia* and *Le. maculans* (Frantzeskakis et al., 2019) where combined TE and RIP activity led to the formation of a stable core compartment associated with house-keeping functions, and an accessory compartment that is more variable and highly dynamic (Frantzeskakis et al., 2019). By contrast, the genomes of most Pezizomycotina that no longer use RIP show less obvious genome compartmentalization (Dong et al., 2015; Frantzeskakis et al., 2019; Müller et al., 2019). Therefore, the availability of genome-wide RIP data may in future prove valuable to understanding such trends in genome evolution.

The data presented here suggest that the rigor of genome-wide RIP analyses is dependent on the quality of the genome data being examined. Much of the recorded RIP was in the repeat-rich regions of genome assemblies. Many of these regions represent the telomeric, centromeric regions of chromosomes, and regions with a high frequency of TEs and repeat sequences. However, these genomic regions are often absent or fragmented from low quality genome assemblies (Testa et al., 2016). Accordingly, low-quality, low-coverage, and short read assemblies typically do not capture the genomic regions extensively affected by RIP (Testa et al., 2016). Therefore, care should be taken when interpreting genome-wide RIP statistics because low-quality genome assemblies may lead to an underestimation of the true extent of RIP on a genome-wide scale, although it can provide preliminary insight on the occurrence of RIP in the fungus in question.

The work presented in this study reports and summarizes the extent and occurrence of RIP by using a set of criteria to describe different Classes (1–6) of RIP. Although the use of these categories greatly simplified and streamlined our genome comparisons, separation of genomes based purely on the content of particular types of mutation may be misleading. However, as more information becomes available, the RIP classes delineated here may be revised and refined to improve their informativeness. For instance, for fungi with fragmented and/or incomplete genome assemblies, the true extent of RIP may remain underestimated until superior genome assemblies become available. Secondly, as each genome investigated in this study represents a single strain from a single population, the analysis of multiple isolates will allow for comparison of RIP classes within and across species. Thirdly, the exact molecular mechanism underlying RIP has not yet been fully determined, and conclusions drawn on the distribution of RIP-associated genes and the extent of RIP can thus be refined further as more genes are characterized and identified. Also, the effect size of each gene has not yet been quantitatively investigated, which may provide further insight on the molecular mechanisms underlying the occurrence and extent of RIP in fungi. For instance, some representatives of the Taphrinomycotina have detectable homologs for many of the genes associated with RIP, yet evidence of RIP in these fungi are lacking. Therefore, functional investigations such as expression analyses and functional gene knockouts might provide greater insight on the RIP capability of the fungi investigated in this study.

In conclusion, genome-wide RIP data for representatives of the Ascomycota have provided a comprehensive view of the occurrence, extent, and distribution of RIP. Data generated using a sliding window-based method yielded superior results compared to those generated using TE sequence alignments. Also, application of these sliding window-based analyses, where all parameters were considered within the same genetic context, reduced the occurrence of erroneous identification of RIP. It enabled the evaluation of the extent and occurrence of RIP for each sequenced representative investigated using a uniform approach. The results of this study have further provided the first reported evidence of RIP in some fungi and for sequenced representatives displaying previously unknown traces of ancestral RIP. Additionally, this work provided information on RIP from fungi representing varying degrees of divergence from the ancestor of the Ascomycota, thus allowing for conclusions to be drawn regarding the taxonomic range and evolutionary history of RIP. Previous work lacked a uniform method to described observed variation of RIP between different species. Here, we presented six categories to summarize and describe the variation in extent of RIP observed for these fungi. The body of knowledge generated in this study forms a baseline to enable future comparative analyses of RIP of newly sequenced genomes. Moreover, the data generated here can serve as a basis for functional investigations to address questions relating to the biological and molecular determinants directing the observed variation in RIP of the Ascomycota.

## Data Availability Statement

The original contributions presented in the study are included in the article/Supplementary Material, further inquiries can be directed to the corresponding author.

## Author Contributions

SW, ES, LV, NM, and BW: conceptualization, methodology, writing – review, and editing. SW, ES, BW, and NM: software. SW, ES, and LV: validation and investigation. SW and LV: formal analysis. ES, LV, and BW: resources. SW, ES, LV, and BW: data curation. SW and ES: writing – original draft preparation, critical revisions, and writing. SW: visualization. ES, LV, NM, and BW: supervision. ES: project administration. BW and ES: funding acquisition. All authors contributed to the article and approved the submitted version.

### Conflict of Interest

The authors declare that the research was conducted in the absence of any commercial or financial relationships that could be construed as a potential conflict of interest.
