# Supplementary material for: Genome-Wide Analyses of Repeat-Induced Point Mutations in the Ascomycota
Source: Front Microbiol. 2021 Feb 1;11:622368. doi: 10.3389/fmicb.2020.622368 (PMC7882544; doi:10.3389/fmicb.2020.622368)

Figure S1:

Figure S1.1: Summary of genome-wide RIP analyses of Ascomycota fungi investigated in this study. The first panel represents a cladogram that summarizes the phylogenetic relationships among the fungi investigated, with the microsporidian fungus *En. cuniculi* serving as the root. Notations following species names include an asterisk which denotes which those species where a chi-square test indicated statistical significance, indicating the frequency of RIP-positive windows in the simulated data and that of the genome sequences investigated differed significantly (P<0.01 confidence level); the letters "A", "S", and "U", indicate asexual, sexual and unknown, respectively; percentages (%) of predicted repetitive content. The heatmap illustrates the total proportion (%) of each genome assembly that constitutes RIP mutations and Large RIP Affected Regions (LRARs). The final panels (left) indicate the presence/absence in each genome of the RIP-associated genes (Table S3). The colour coding used in the different panels is explained in the keys provided.

RIP analyses of control organisms. Figure S1.2.1-Figure S1.2.6. depicts the results of the RIPCAL analyses for each of the control organisms. Panel A illustrates RIPCAL alignment-based analyses, B changes in RIP index values and C the changes in point mutations recorded across the length of the alignment Summary of the genetic features of the negative control organisms. Figure S1.3 illustrates a summary of the genetic features of the chromosome assemblies of the control organisms ([Fig.S1.3.A] *En. Cuniculi*, [Fig.S1.3. B] *Ca. albicans*, C] *Tr. Reesei* and D] *Le. maculans* [ten largest scaffolds]) investigated in this study. For each panel changes in RIP composite index values[1] are illustrated (values greater than 0 indicate RIP) changes in the frequency of TE distribution and changes in GC content are indicated in the second and third panels, respectively. The data for *E. coli* is presented in Figure 2 and the results of the *N. crassa* genome is presented in Figures S2..

[1]These values reflect changes in RIP composite index values and does not consider the additional parameters of RIP substrate and RIP product index values (van Wyk *et al*. 2019a). A full list of genome-wide RIP statistics, implementing stringent parameters, are available for these organisms in Table S5.

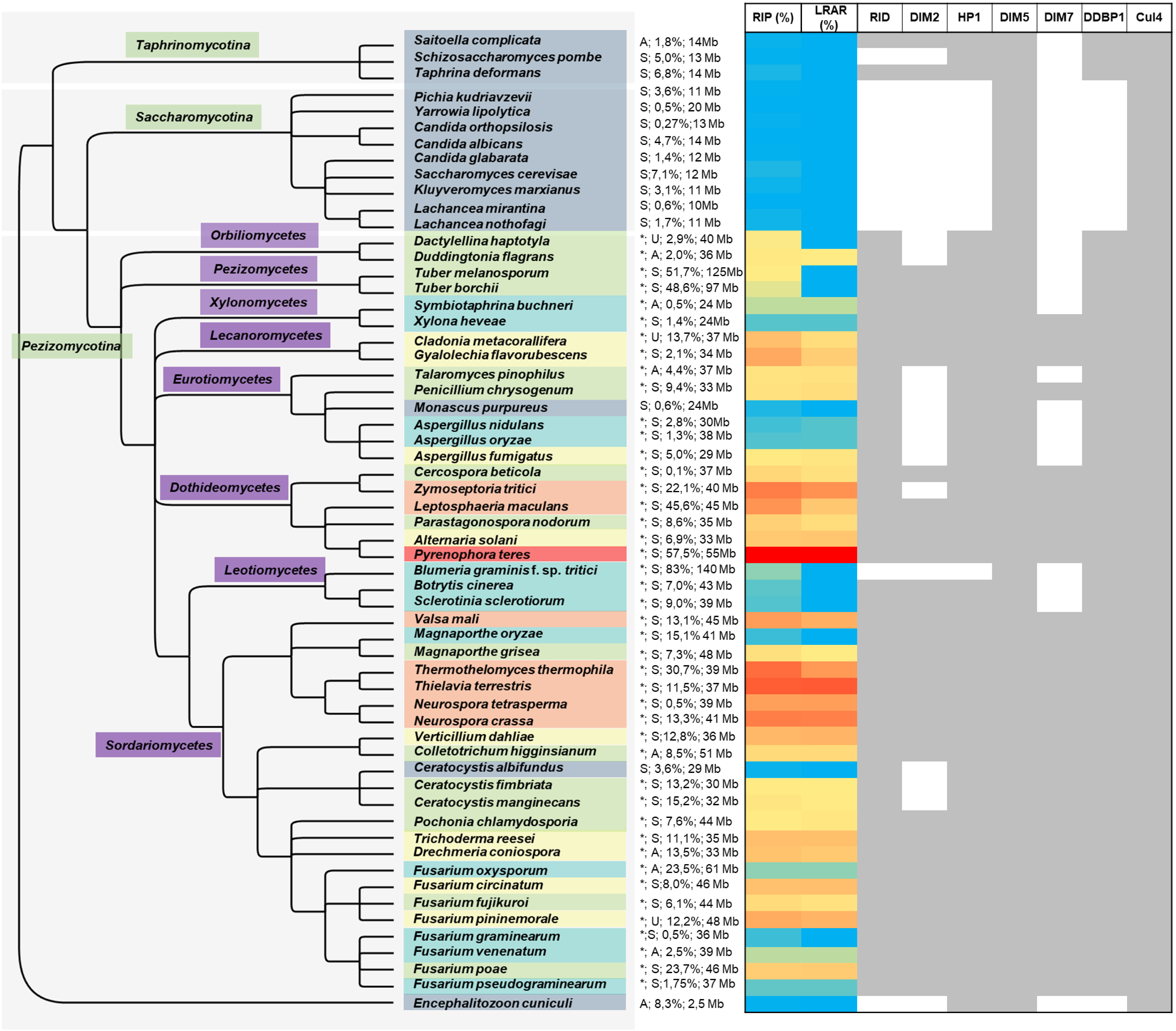

RIP class					
Class 1 (0.0%-<0.2%)	Class 2 (0.2%-<1.0%)	Class 3 (1.0%-<5.0%)	Class 4 (5.0%-<10.0%)	Class 5 (10.0%-<20.0%)	Class 6 (>20.0%)
No RIP	Low RIP	Moderately low RIP	Moderate RIP	Moderately high RIP	High RIP

Percentage (%)																			
0.00	0.15	0.30	0.45	0.50	0.60	0.70	0.80	0.90	1.00	1.50	2.00	4.00	6.00	8.00	10.00	15.00	20.00	25.00	30.00

Figure S1.2.

1. Encephalitozoon cuniculi

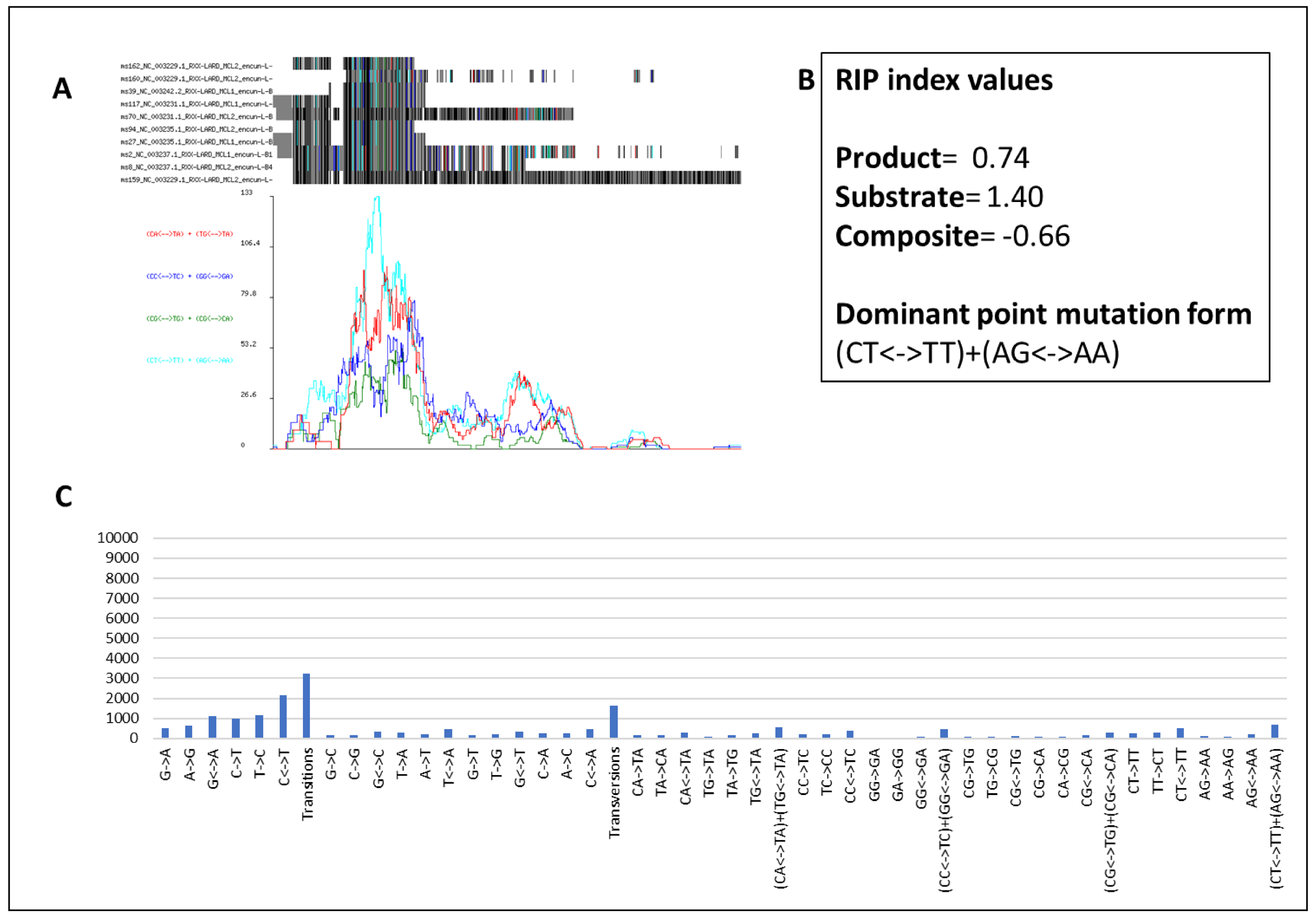

2. Candida albicans

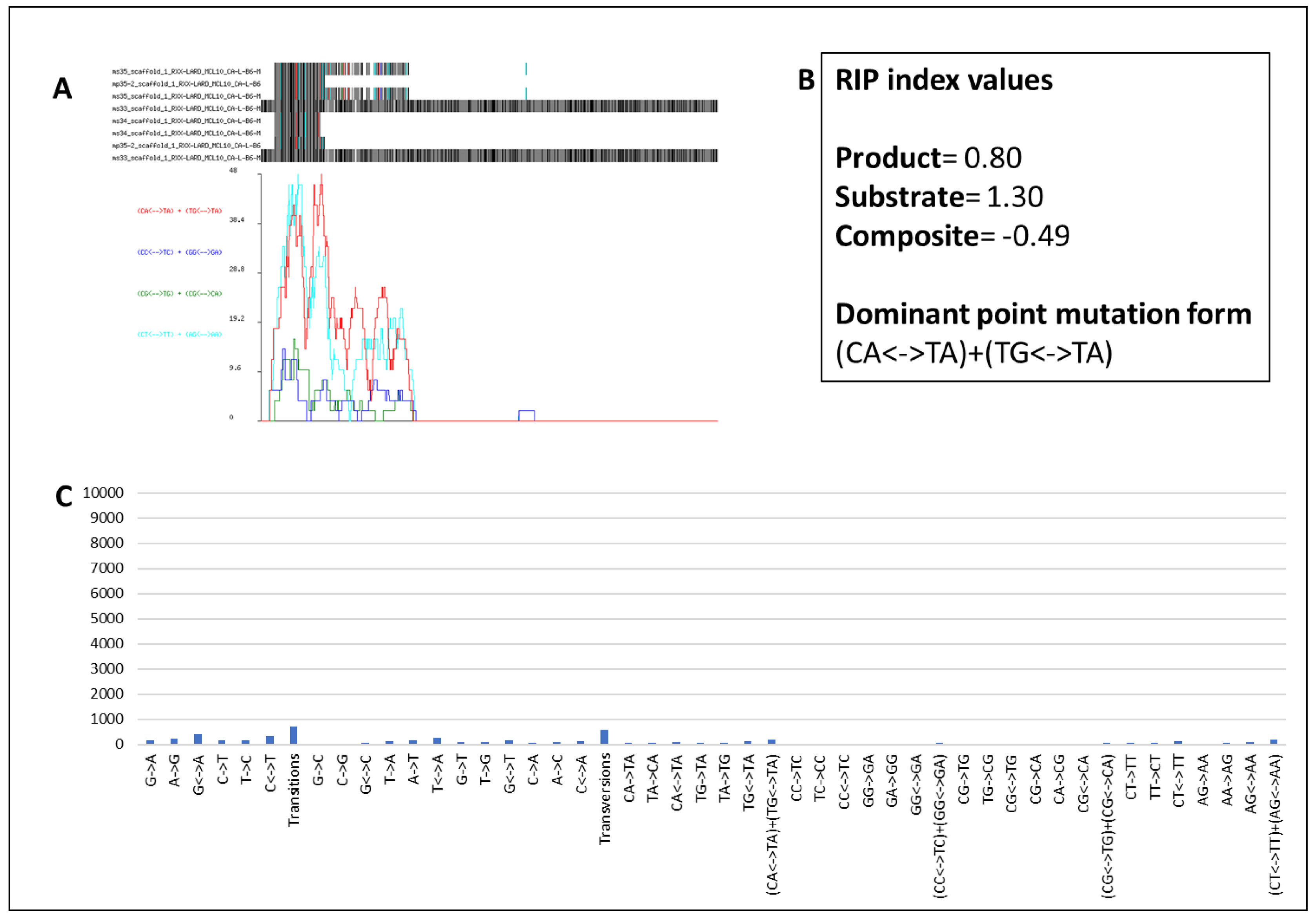

3. Escherichia coli

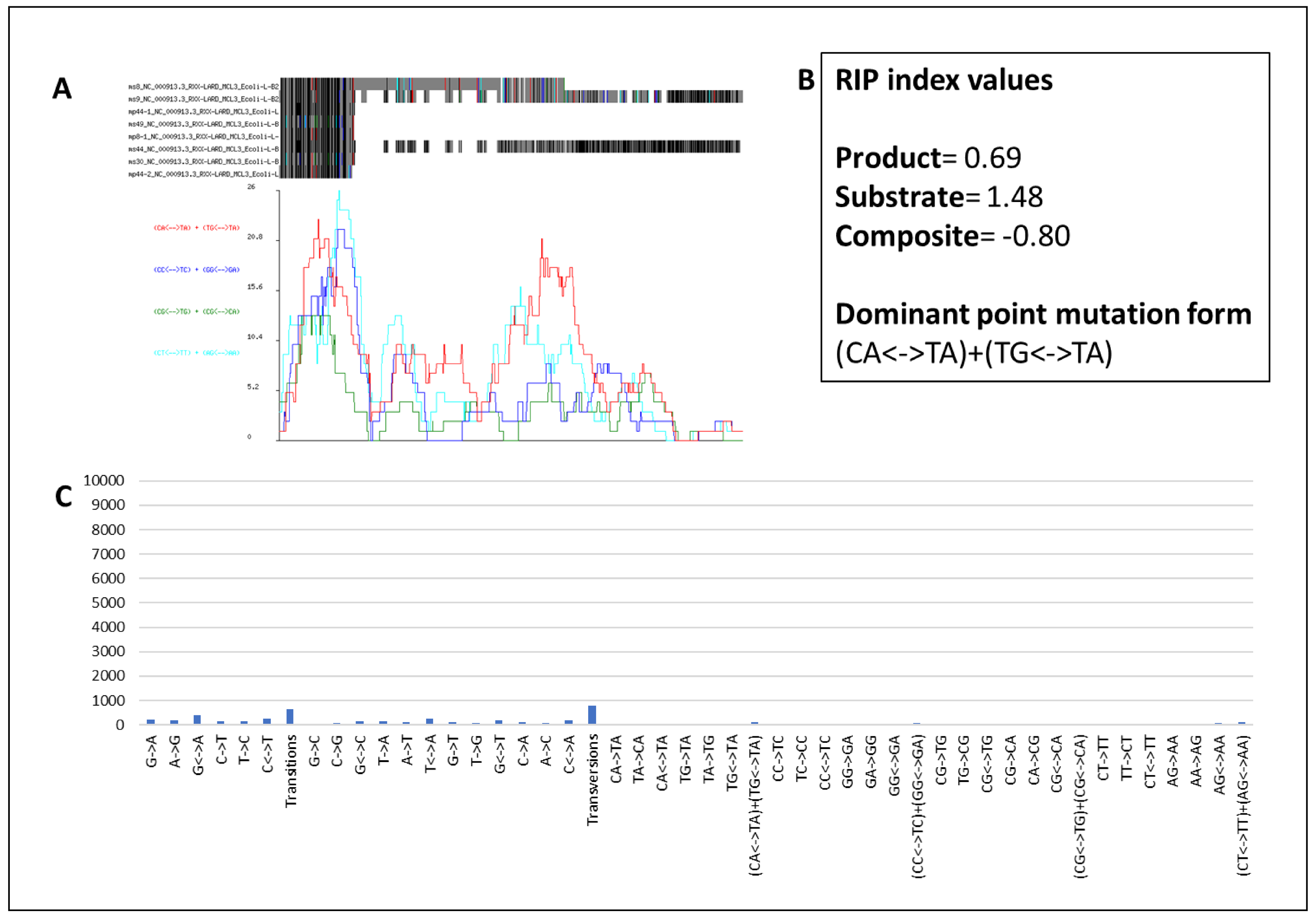

4. Neurospora crassa

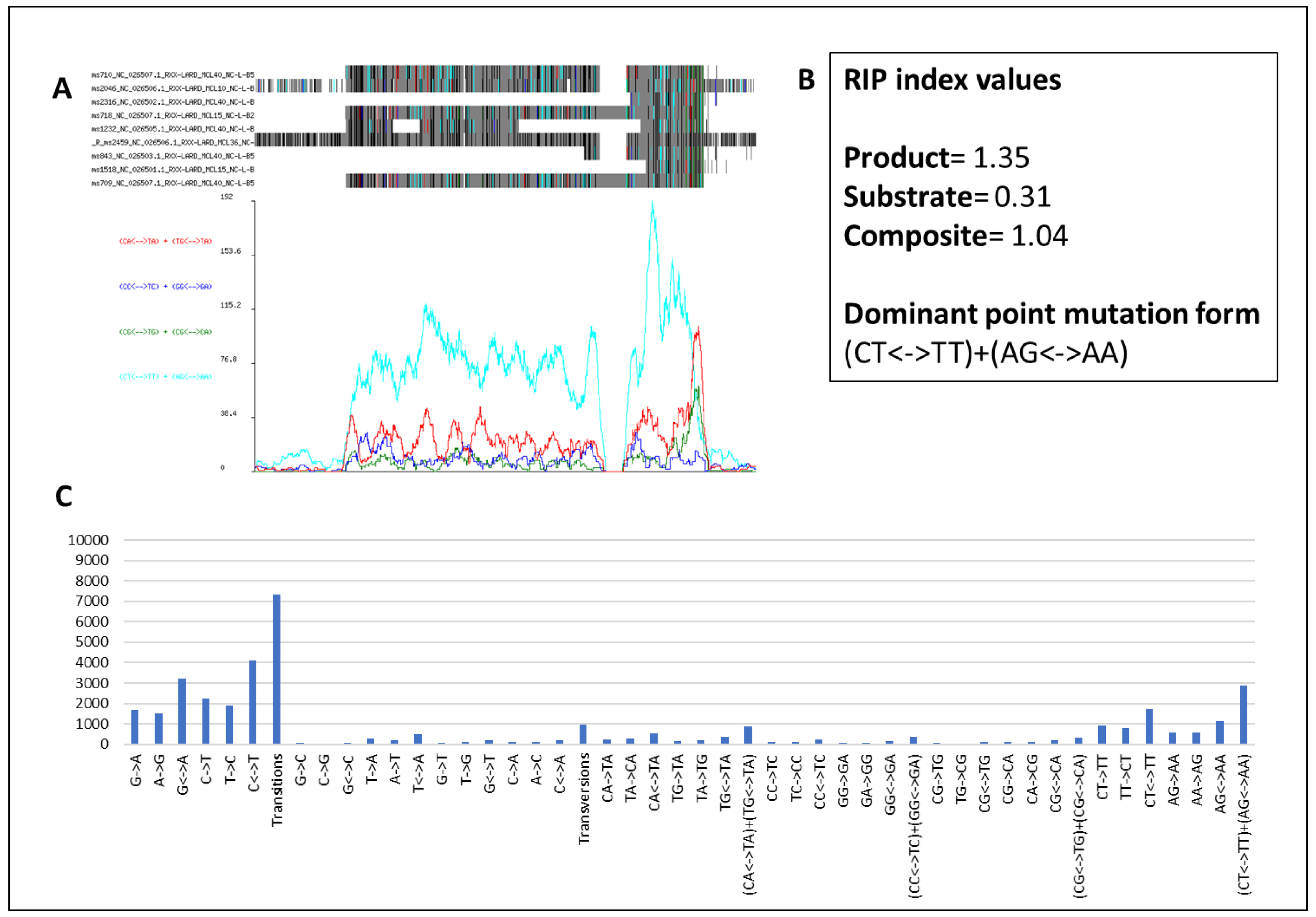

5. Trichoderma reesei

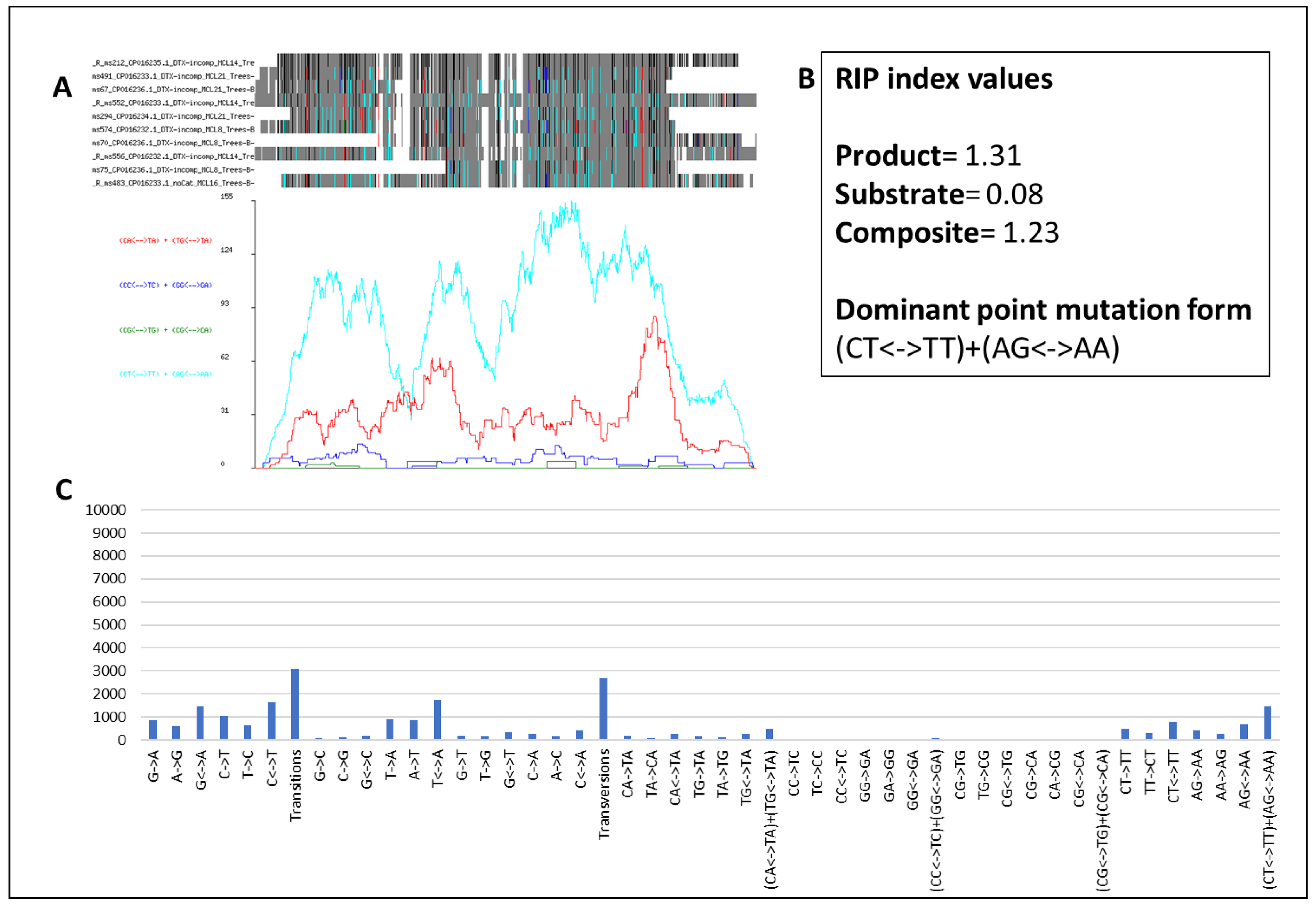

6. Leptosphaeria maculans

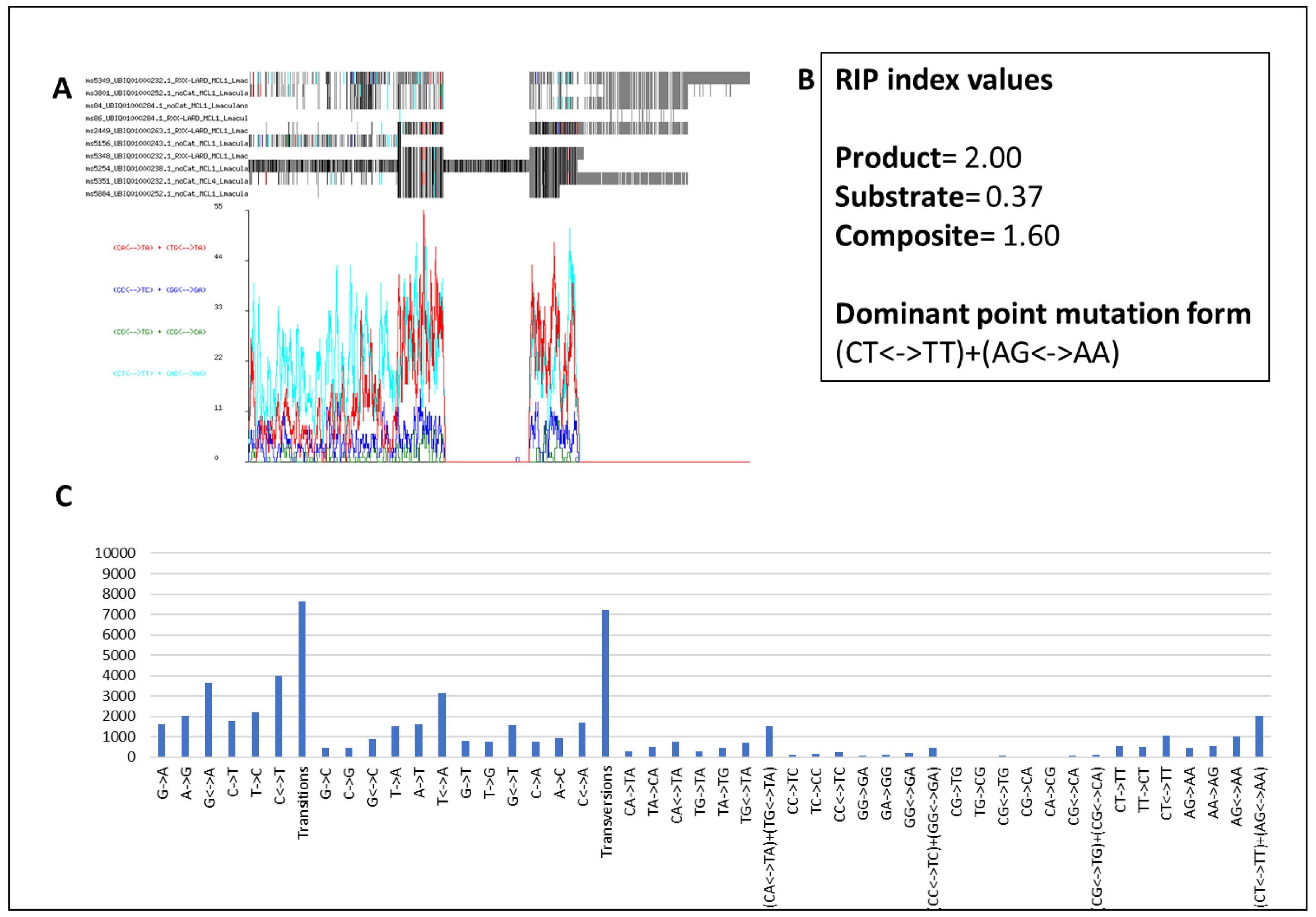

Figure S1.3. A

Encephalitozoon cuniculi

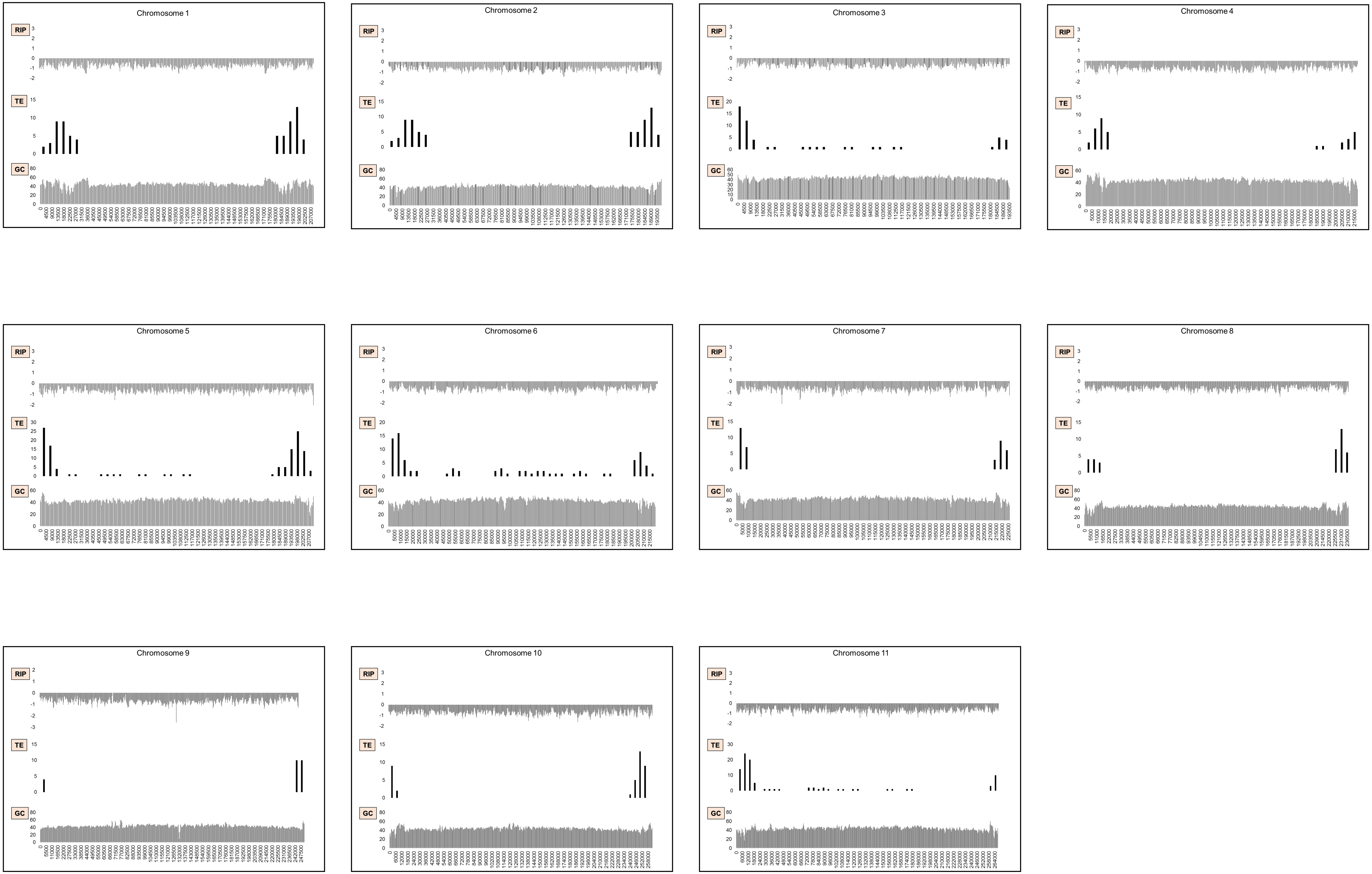

Figure S1.3. B

Candida albicans

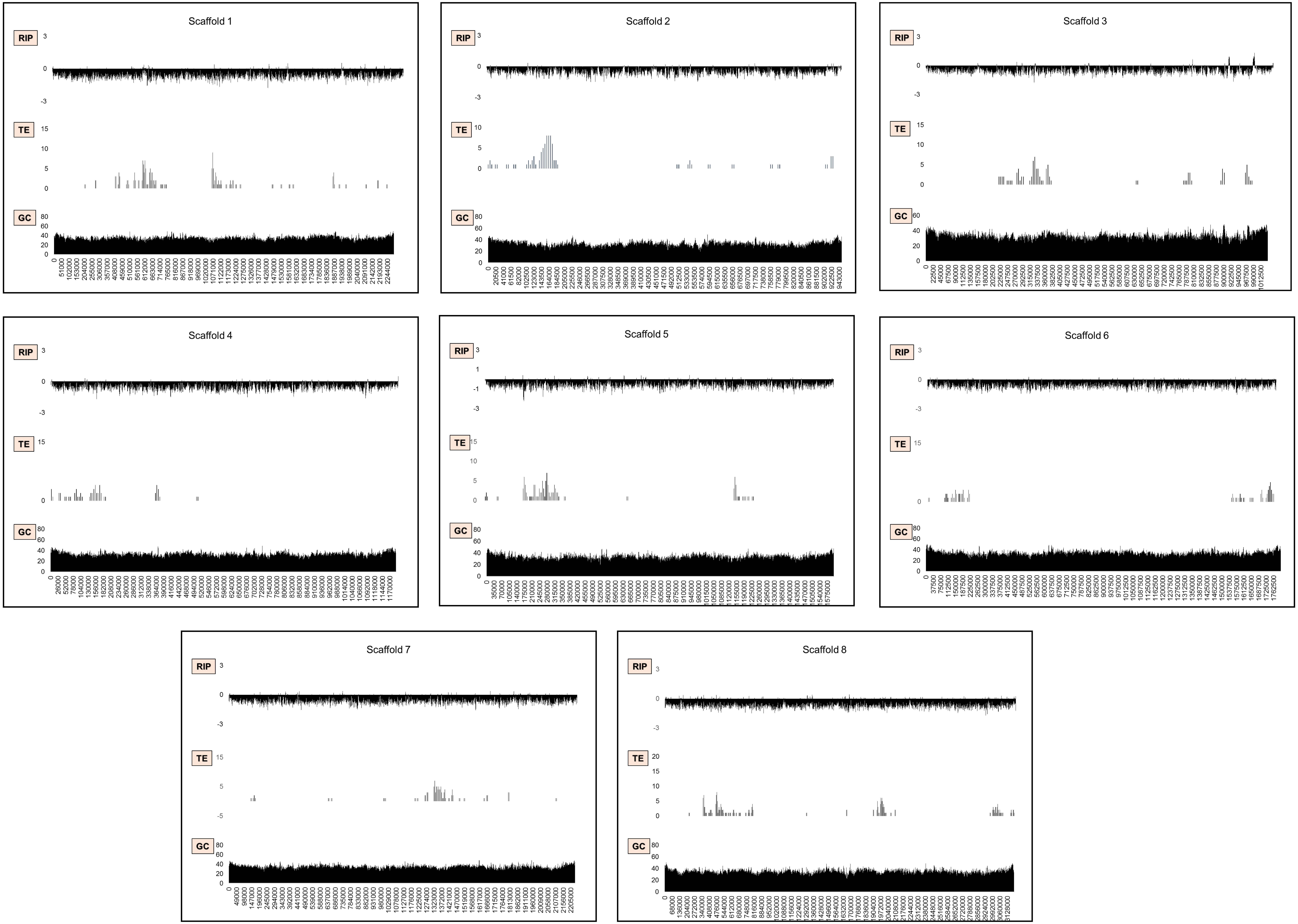

Figure S1.3. C.

Trichoderma reesei

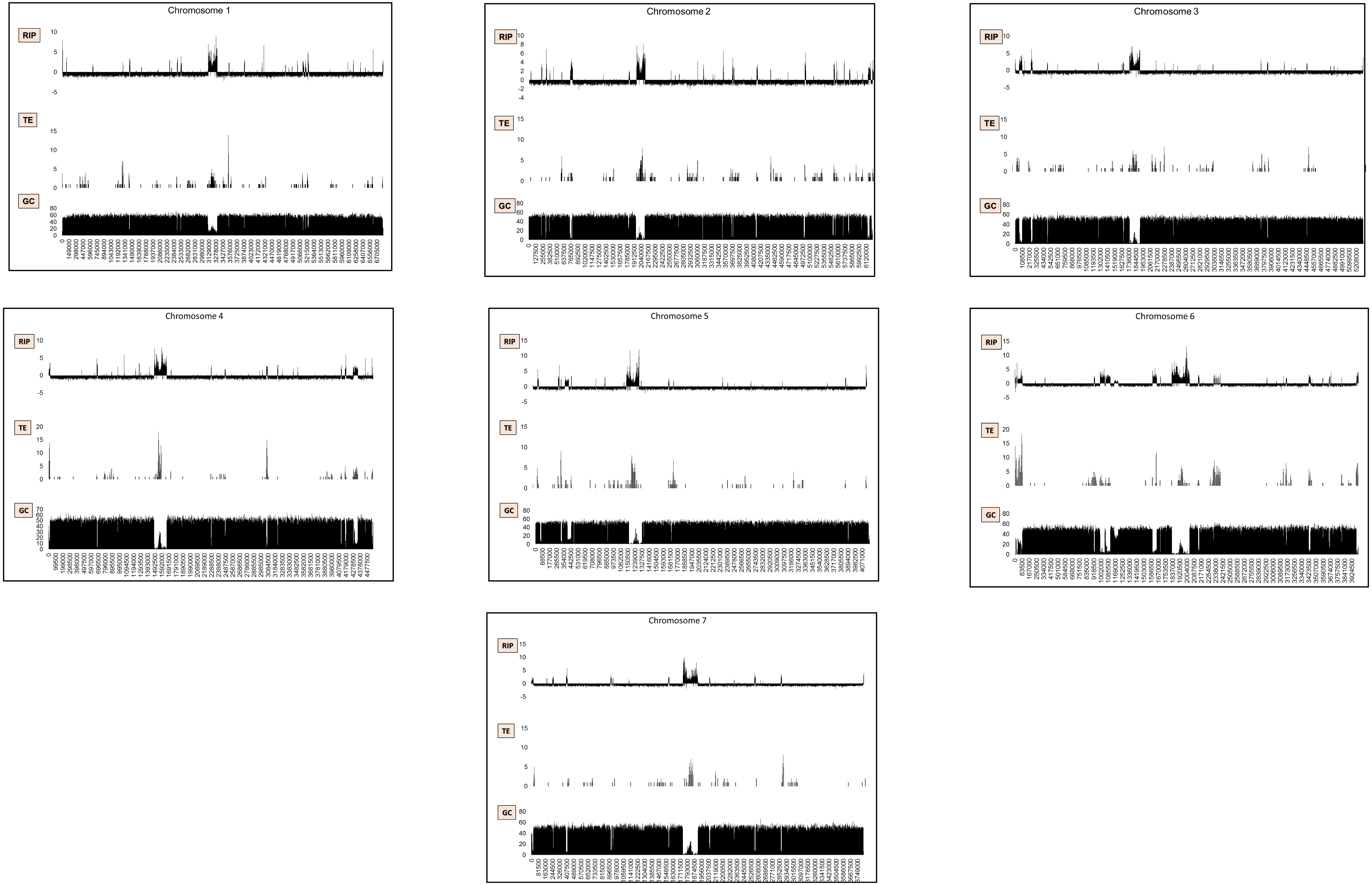

Figure S1.3. D

Leptosphaeria maculans

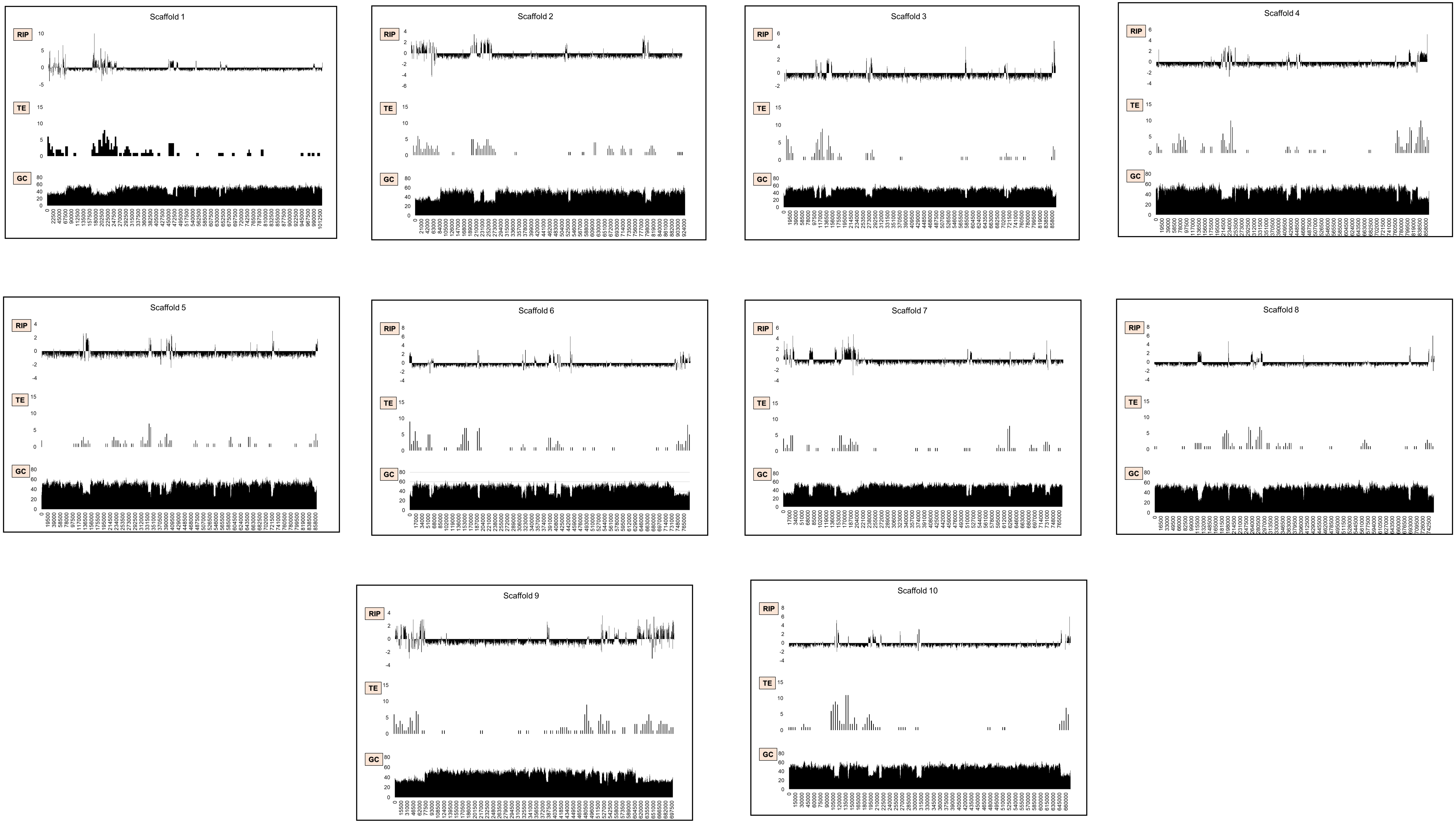

Supplement: Supplementary file 1 [file Data_Sheet_1.zip › Figure S1.pdf]
