# Supplementary material for: Genome-Wide Analyses of Repeat-Induced Point Mutations in the Ascomycota
Source: Front Microbiol. 2021 Feb 1;11:622368. doi: 10.3389/fmicb.2020.622368 (PMC7882544; doi:10.3389/fmicb.2020.622368)
Supplement: Supplementary file 1 [file Data_Sheet_1.zip › Figure_S2.pdf]

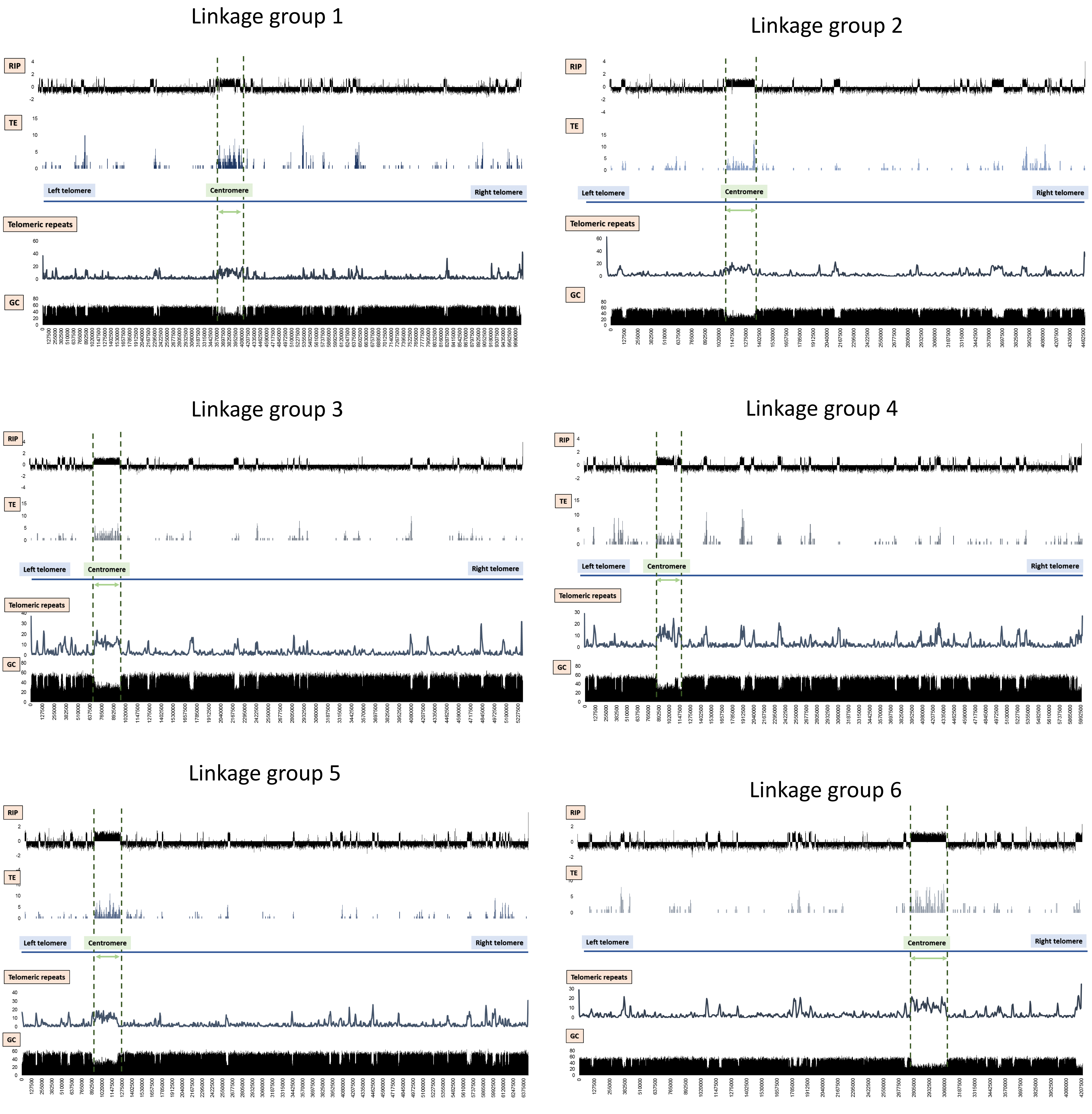

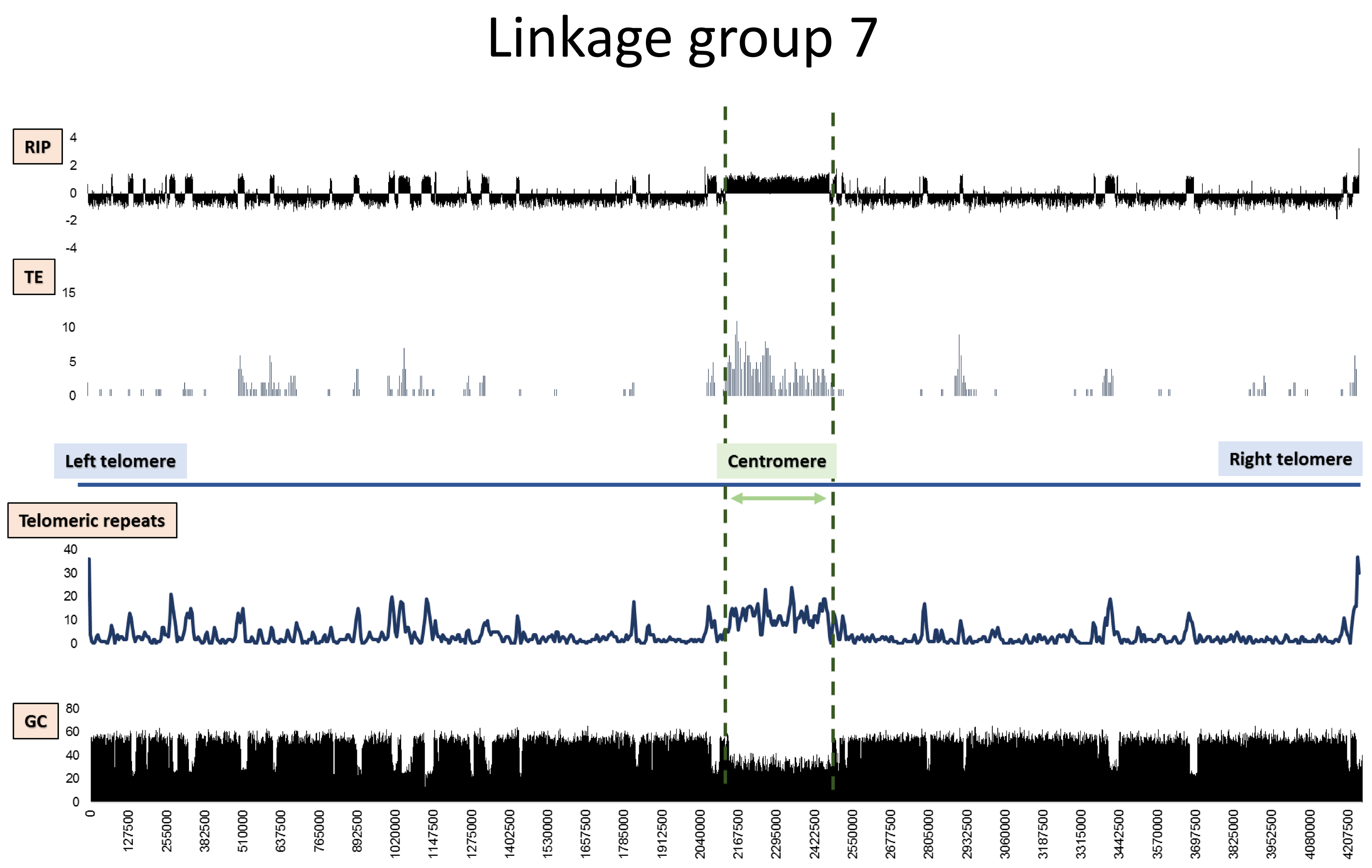

Figure S2: Summary of the genetic features of the individual linkage groups/chromosomes of the assembly of *Neurospora crassa*. The top panel depicts the changes in RIP composite index values across the length of chromosome using a 1 000 base pairs (bp) window and increments of 500 bp. Values above 0 indicate RIP. The second panel depicts the distribution of TEs associated repeated sequences (larger than 100 bp) across the length of the chromosome, (10Kb window and 5Kbp increments). The third panel presents the putative genomic locations of the centromeres and telomeric regions. The fourth panel of each linkage group depicts a summary of changes in the total count of telomeric repeat sequences (TTAGGG/CCCTAA) calculated for every 10Kbp windows and 5Kbp increments. The bottom panel illustrates the changes in GC content calculated 1 000bp window and 500bp increments.
